# CyuR is a dual regulator for L-cysteine dependent antimicrobial resistance in *Escherichia coli*

**DOI:** 10.1038/s42003-024-06831-0

**Published:** 2024-09-17

**Authors:** Irina A. Rodionova, Hyun Gyu Lim, Ye Gao, Dmitry A. Rodionov, Ying Hutchison, Richard Szubin, Christopher Dalldorf, Jonathan Monk, Bernhard O. Palsson

**Affiliations:** 1 Department of Bioengineering, Division of Engineering, University of California San Diego, La Jolla, CA USA; 2 Department of Biological Sciences and Bioengineering, Inha University, Incheon, Korea; 3 The Second Hospital of Shandong University, Jinan, Shandong PR China; 4 Sanford-Burnhams-Prebys Medical Discovery Institute, La Jolla, CA USA; 5 Department of Pediatrics, University of California San Diego, La Jolla, CA USA; 6 Novo Nordisk Foundation Center for Biosustainability, Technical University of Denmark, Lyngby, Denmark

**Keywords:** Gene expression, Infection

## Abstract

Hydrogen sulfide (H_2_S), mainly produced from L-cysteine (Cys), renders bacteria highly resistant to oxidative stress and potentially increases antimicrobial resistance (AMR). CyuR is a Cys-dependent transcription regulator, responsible for the activation of the *cyuPA* operon and generation of H_2_S. Despite its potential importance, its regulatory network remains poorly understood. In this study, we investigate the roles of the CyuR regulon in a Cys-dependent AMR mechanism in *E. coli* strains. We show: (1) Generation of H_2_S from Cys affects the sensitivities to growth inhibitors; (2) Cys supplementation decreases stress responses; (3) CyuR negatively controls the expression of *mdlAB* encoding a potential transporter for antibiotics; (4) CyuR binds to a DNA sequence motif ‘GAAwAAATTGTxGxxATTTsyCC’ in the absence of Cys; and (5) CyuR may regulate 25 additional genes which were not reported previously. Collectively, our findings expand the understanding of the biological roles of CyuR relevant to antibiotic resistance associated with Cys.

## Abstract (web summary)

CyuR, an L-cysteine-dependent transcription regulator, has various roles in antimicrobial resistance such as generating hydrogen sulfide, mitigating stress, and activating a potential growth inhibitor efflux pump.

## Introduction

The emergence of antimicrobial-resistant (AMR) bacteria has been recognized as a serious threat to public health^1^. Bactericidal antibiotics (ABx, e.g., quinolones, β-lactams) generally increase the level of hydroxyl radicals resulting in lethal oxidative stress to cells^2^. It is known that hydroxyl radicals are produced from damaged key metabolic pathways including the TCA cycle, depletion of NADH, inactivated Fe-S clusters, and stimulation of the Fenton reaction^3^. Accordingly, in order to survive in the presence of ABx, bacteria utilize diverse resistance mechanisms that include changing the expression of genes responsible for oxidative stress mitigation and efflux of an ABx.

Recently, the production of hydrogen sulfide (H_2_S) was suggested to be one of the key mechanisms for increased tolerance against many antibiotics^4,5^. Many bacteria can generate H_2_S via catabolism of L-cysteine (Cys) or reduction of an inorganic sulfur source, such as thiosulfate. It was shown that H_2_S decreases the amount of intracellular reactive oxygen species (ROS) by reacting with H_2_O_2_, a substrate of the Fenton reaction^6^, and stimulating the gene expression for enzymes that scavenge ROS. Therefore, the generation of H_2_S contributes to the enhancement of intrinsic antibiotic resistance^4^. Additionally, one recent study showed H_2_S can react with cysteine and yield Cys hydropersulfide (CysSSH), which inactivates multiple antibiotics such as penicillin G, ampicillin, and meropenem^7^. These reports suggest that Cys-derived H_2_S plays a critical role in antibiotic resistance.

In *E. coli*, CyuR, also known as YbaO or DecR, was reported to be an important regulator for the generation of hydrogen sulfide from Cys in anaerobic conditions^8,9^. Its physical interaction with the *cyuPA* operon consisting of *cyuA*, previously referred as *yhaN* or *yhaM*, and *cyuP* (b3110, also referred as *dlsT* or *yhaO*) was reported by two independent studies that utilized systematic evolution of ligands by exponential enrichment^8^ (SELEX) and chromatin immunoprecipitation exonuclease (ChIP-exo) sequencing^10^, respectively. *E. coli* has at least six enzymes (CyuA, CysM, CysK, MetC, DcyD, TnaA) that have Cys desulfhydrase activity to generate H_2_S from the decomposition of Cys to pyruvate and ammonium^9,11^. Among them, CyuA is suggested to be a major enzyme for H_2_S production in anaerobic conditions in *E. coli*^8,9,12^. On the other hand, *cyuP* was shown to encode an importer for Cys or serine^9,12^. In *Yersinia ruckeri* the homologs of *cyuP and cyuA* are encoded in one operon - *cdsAB* - and involved in Cys uptake; additionally, the operon was shown to be critical for full virulence of *Y. ruckeri* in fish^13^. However, despite the importance of CyuR, much of its detailed regulatory information (e.g., its binding motif) and effects of Cys are not well characterized and thus remain unknown.

In this study, we report the effects of Cys and the detailed regulatory roles of CyuR on antimicrobial resistance in *E. coli*. First, we utilized phenotype microarray plates to study the effects of Cys on a broad spectrum of antibiotics and growth inhibitors (hereafter collectively referred to as antibiotics) with *E. coli* laboratory strain W and three clinical isolates (GN02094, GN02148, and GN02007). Second, the role of CyuR in Cys metabolism was investigated by comparing the resistance of the wildtype *E. coli* K-12 MG1655 and its *cyuR*-deletion mutant. The regulatory network of CyuR was reconstructed by investigating the clustering information of co-expressed genes (i.e., iModulons) obtained by an independent component analysis (ICA) of multiple *E. coli* gene expression profiles^14,15^. Lastly, we show that CyuR regulation is widely conserved in many Enterobacterial genomes and for the first time report the binding motif of CyuR, enabled by genome-wide motif scanning, to predict additional target genes. Collectively, our results suggest that the elucidated CyuR-mediated Cys-dependent AMR mechanism is widely conserved across species and Cys supplementation should thus be considered carefully during the treatment of AMR bacteria.

## Results

### The presence of Cys increases antimicrobial resistance in laboratory and clinical *E. coli* strains

**Fig. 1:**
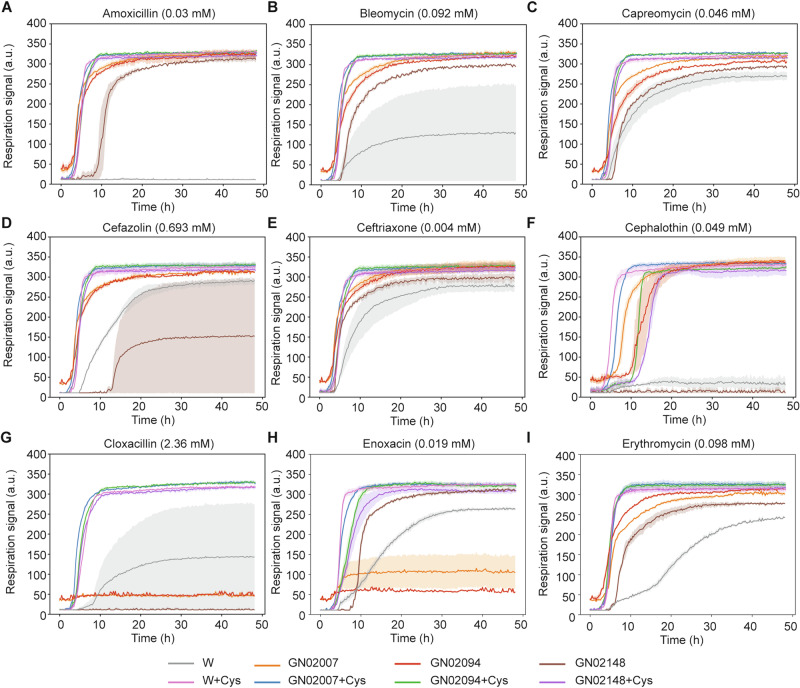
Cys supplementation increases antimicrobial resistance in *E. coli.* **A**–**I** Respiration signal of *E. coli* wildtype W and three clinical isolates, GN02007, GN02094, and GN02148 in the absence and presence of 5 mM Cys with diverse antibiotics contained in phenotype microarray plates. **A** amoxicillin (0.03 mM), **B** bleomycin (0.092 mM), **C** capreomycin (0.046 mM), **D** cefazolin (0.693 mM), **E** ceftriaxone (0.004 mM), **F** cephalothin (0.049 mM), **G** cloxacillin (2.36 mM), **H** enoxacin (0.019 mM), **I** erythromycin (0.098 mM). In all conditions, respiration signals were high when Cys was supplemented. Results for the full antibiotics are summarized in Table S5. The experiments were performed in biological duplicate and shadow indicates the maximum and minimum value of each measurement.

We investigated the effect of Cys on antimicrobial resistance in *E. coli* by using phenotype microarray (PM) plates. As a test case, we chose *E. coli* W and three clinical isolates, GN02094, GN02148, and GN02007, from the Duke Bloodstream Infection Biorepository (Table S1)^16^. The three clinical *E. coli* strains were chosen because they were collected from bodily fluids of deceased people and their genome sequences are available. *E. coli* W is known to be more resistant against antibiotics^17,18^ and acids^19,20^; more importantly, the W strain belongs to a relatively closer phylogroup to the three clinical isolates than other laboratory *E. coli* strains (Table S2). Additionally, the desulfhydrase activity in *E. coli* W appeared to be more comparable to the activities in the three clinical strains and higher than the activity in the *E. coli* K-12 MG1655 and BW25113 strains in the presence of 5 mM Cys (Fig. S1). We grew these strains in PM11C and PM12B plates, containing 48 antibiotics at four different concentrations (Tables S3 and S4) and monitored a respiration signal, as the mean of viability of cells, in the presence and absence of 5 mM Cys (Fig. 1, Table S5, and Supplementary Data 1). Although there were variations depending on strains, notably, Cys supplementation increased respiration signals of all of these strains for 21 antibiotics (44% of the tested 48 antibiotics): L-aspartic-β-hydroxamate, chloramphenicol, cloxacillin, colistin, demeclocycline, enoxacin, erythromycin, lincomycin, lomefloxacin, minocycline, nafcillin, ofloxacin, penimepicyclin, potassium tellurite, D,L-serine hydroxamate, spectinomycin, spiramycin, sulfadiazine, sulfamethazine, tobramycin, and vancomycin. Except for three antibiotics, 5-fluoroorotic acid, polymyxin B, and sisomicin, increased respiration signals were observed when Cys was supplemented for the remaining antibiotics in at least one strain. These observations clearly indicate enhanced antimicrobial resistance by Cys supplementation.

### Transcriptomic analysis unveiled that Cys supplementation decreased stress responses in *E. coli*

**Fig. 2:**
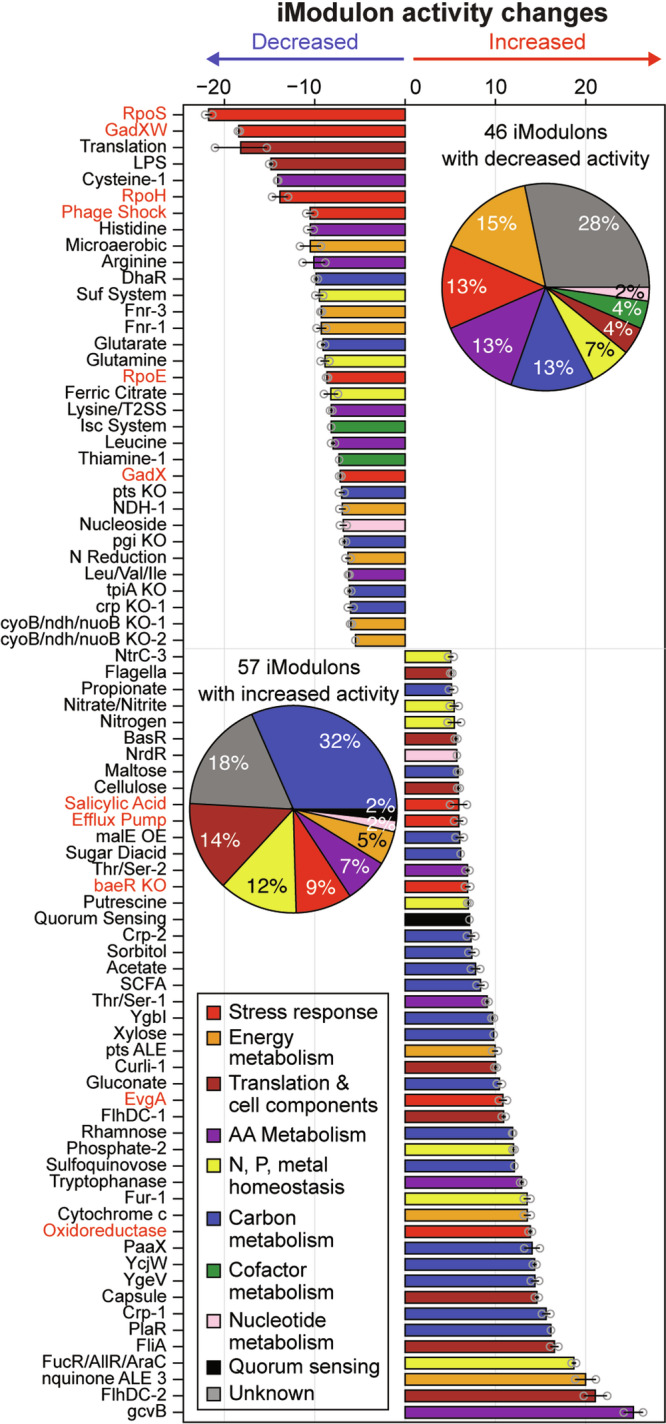
iModulon activity changes by Cys supplementation. Inferred iModulon activity changes by 5 mM Cys supplementation. The names of stress-related iModulons are colored in red. The two inset pie charts show the number of functions for iModulons with changed activities. ‘Other’ iModulons were not shown in the bar graph. A full list of iModulons and activities are in Table S6. The iModulon activities were inferred from gene expression profiles of two biological duplicates and data points indicate the two values. Bar colors: red, stress response; orange, energy metabolism; brown, translation & cell components; purple, amino acid (AA) metabolism; yellow, N, P, metal homeostasis; blue, carbon metabolism; green cofactor metabolism; pink, nucleotide metabolism; black, quorum sensing; grey, unknown.

To gain an understanding about the increased resistance in response to Cys, we investigated differences in the transcriptome with Cys supplementation in *E. coli* W treated with ampicillin by analyzing iModulon activity changes (Fig. 2 and Supplementary Data 1). Although the conventional differential expression gene (DEG) analysis is useful, we found 3176 genes (66.7%) out of the 4764 annotated genes in *E. coli* with expression fold changes greater than 2 (*p*_adj_ < 0.05, Fig. S2), challenging its comprehensive understanding. Alternatively, iModulons are groups of co-expressed genes, identified by performing an ICA, a machine-learning algorithm for a compendium of gene expression profiles of a given microorganism^14,21^. It was shown that comparing iModulon activities for a dataset is effective for interpreting complex transcriptome changes rather than analyzing the individual gene expression levels with a reduced dimensionality^15,22^. For *E. coli*, a recent study identified 201 iModulons from 1035 high-quality gene expression profiles, collected under 533 conditions^14,21^. Indeed, with this iModulon structure, nearly half of the total variance (45.5%) in gene expression was explained and we observed that 103 iModulons had changed activities greater than 5 by the Cys supplementation (Table S6); the activities of 46 and 57 iModulons decreased and increased, respectively.

From the changed iModulon activities, the effect of Cys supplementation could be inferred. Notably, many iModulons with decreased activities include stress-response related iModulons (e.g., “RpoS” for global stress, “GadXW” for acid stress, and “RpoH” for thermal stress). This observation showed that the Cys supplementation greatly protects bacteria against diverse stress imposed by antibiotics therefore increasing microbial tolerance. Additionally, decreased activities were observed for amino acid metabolism-related iModulons (e.g., “Cysteine-1”, “Arginine”, “Histidine” for their biosynthesis) and energy metabolism-related iModulons (e.g., “microaerobic”, “Fnr-1”). On the other hand, many iModulons with increased activities were related to amino acid metabolism (e.g., “gcvB”), extracellular components (e.g., “FlhDC-1”, “FlhDC-2”, and “FliA-1” for flagella, “Curli-1” for curli, “RcsAB” for colanic acid capsule, “EvgA” for the efflux pumps regulation), and carbon metabolism (e.g., “PlaR” for diketo-L-gulonate, “PaaX” for phenylacetate^23^, “YgeV” for uric acid^24^, “YcjW” for carbohydrate utilization^25^). Although detailed regulatory mechanisms are not clear and thus need further studies, it was inferred that Cys supplementation promotes gene expression related to cellular motility and carbon catabolism, while reducing the expression of stress-related iModulons.

### Inactivation of CyuR sensitizes *E. coli* to diverse antibiotics

**Fig. 3:**
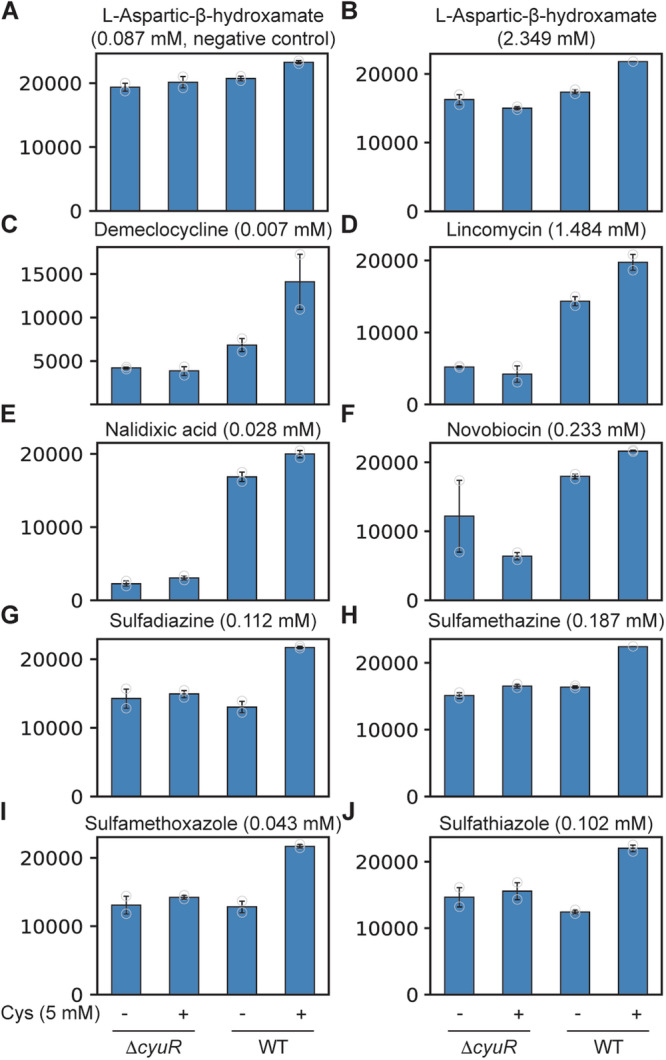
The essentiality of CyuR for Cys-involved antimicrobial resistance. **A**–**J** Respiratory signal (arbitrary unit, a.u.) of *E. coli* K-12 MG1655 and its *cyuR*-deleted mutant grown in a Biolog plate with nine different antibiotics: **A** L-aspartic-β-hydroxamate 0.087 mM (negative control), **B** L-aspartic-β-hydroxamate 2.349 mM, **C** demeclocycline 0.007 mM, **D** lincomycin 1.484 mM, **E** nalidixic acid 0.028 mM, **F** novobiocin 0.233 mM, **G** sulfadiazine 0.112 mM, **H** sulfamethazine 0.187 mM, **I** sulfamethoxazole 0.043 mM, **J** sulfathiazole 0.102 mM. *y*-axis indicate respiration signal (a.u.). Biolog plates (PM11C and PM12B) contain antibiotics at four different concentrations (Supplementary Tables 3 and 4).

We investigated the role of CyuR, which is known to be the major regulator for H_2_S production from Cys, in antibiotic resistance. The expression of its known target gene, *cyuA*, was highest among the six cysteine desulfhydrase genes when Cys was supplemented in the medium (Fig. S3). We compared respiration signals of MG1655 Δ*cyuR*^10^ (Table S1) and its parental strain, MG1655, with and without 5 mM Cys supplementation in PM11C and PM12B plates (Fig. 3 and Supplementary Data 1). Similar to the *E. coli* W strain, the MG1655 strain showed increased respiratory signals when Cys is present for 17 of the 48 tested antibiotics (35%). Although Cys supplementation was effective with a smaller number of antibiotics than those effective for *E. coli* W, this observation supports stress reduction by the generation of H_2_S.

Notably, such improvements by Cys supplementation were lost after deleting *cyuR* in the MG1655 strain for at least nine antibiotics (demeclocycline, L-aspartic-β-hydroxamate, lincomycin, nalidixic acid, novobiocin, paromomycin, sulfadiazine, sulfamethazine, sulfamethoxazole, and sulfathiazole, Fig. 3A–J, and Supplementary Data 1), implying the essentiality of CyuR in Cys-involved antimicrobial resistance. A complementation assay for *cyuR* in the deletion mutant was also tested (Table S7) and supported the role of CyuR for the increased resistance to many of the antibiotics (i.e., L-aspartic-hydroxamate, novobiocin, sulfadiazine, sulfamethoxazole). Interestingly, the resistance was decreased by the deletion of *cyuR* itself, even when Cys was not supplemented in some cases (e.g., lincomycin, nalidixic acid). This observation suggested that CyuR is potentially involved in an additional antibiotic resistance mechanism not related to Cys.

### CyuR is a dual regulator for *cyuR*-*mdlAB* and *cyuPA* in response to Cys exposure

**Fig. 4:**
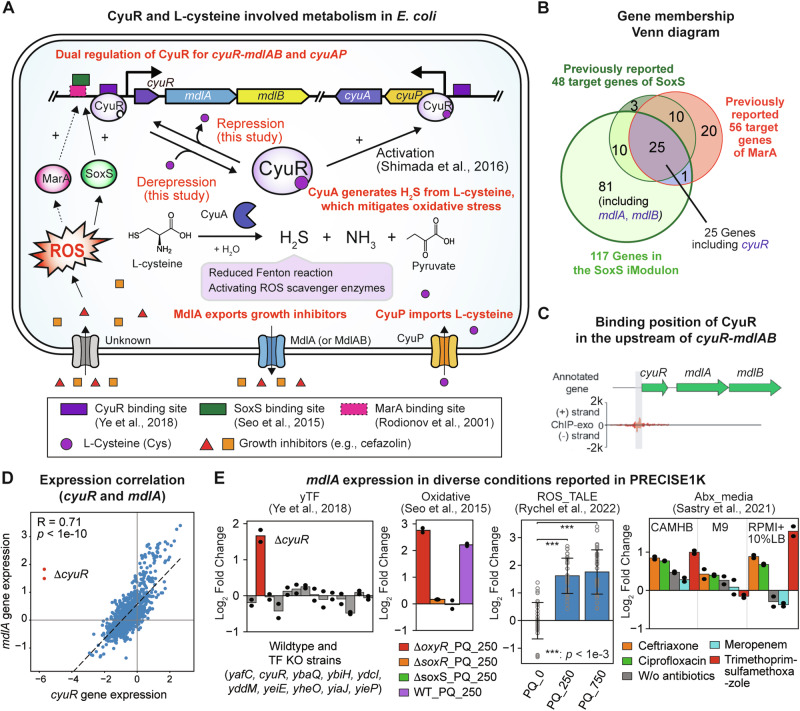
Regulatory information of CyuR and its regulon genes. **A** CyuR (b0447, also called YbaO, DecR) and L-cysteine (Cys) involved metabolism in *E. coli*. CyuR is a dual regulator for *cyuPA*^8^, encoding Cys desulfhydrase and importer, and *mdlAB*, encoding an antibiotic efflux ABC-type transporter MdlA (or MdlAB). In the presence of Cys, Cys-bound CyuR activates the expression of *cyuPA* and derepresses the expression of *mdlAB*. Binding sites of CyuR were obtained from previous studies^8,10^. Degradation of Cys by CyuA generates hydrogen sulfide (H_2_S) which is beneficial to the reduction in Fenton reaction or stimulation of reactive oxygen species scavenging pathway. The expression of *cyuR* is regulated by SoxS^26^ and potentially MarA^27^. **B** A Venn diagram of three gene groups: SoxS iModulon, SoxS regulon, MarA regulon. *cyuR* belongs to the three gene groups; *mdlA* and *mdlB* only belong to the SoxS iModulon; *cyuA* and *cyuP* were not included in any groups. **C** Binding site of CyuR identified from a previous Chip-exo experiment^10^. *y*-axis shows the number of reads mapped to a region. **D** Gene expression correlation between *cyuR* and *mdlA*. Expression of these two genes is highly correlated, supporting that they are co-expressed from the same operon. **E** Gene expression of *mdlA* in multiple projects of PRECISE-1K^15^. The titles of the bar graphs indicate the names of projects that generated gene expression profiles. YTF, a project that characterized the regulons of putative transcriptional factor in *E. coli*^10^; Oxidative, a project that characterized the regulon of OxyR, SoxS, and SoxR^26^; ROS_TALE, a project that studied a wildtype strain and evolved strains tolerized to reactive oxygen species^46^; Abx_media: a project that studied transcriptional responses to multiple antibiotics in different media conditions^47^. Further details were also described in Supplementary Note 1. A two-tailed *t*-test was performed for the ROS_TALE project samples and *p*-values were 1.3 × 10^−15^ (paraquat 0 mM vs 250 mM) and 5.9 × 10^−14^ (paraquat 0 mM vs 750 mM).

Given its potential expanded regulatory roles in antimicrobial resistance, we further carried out a detailed characterization of CyuR. We first collected existing information about the expression of *cyuR* in *E. coli* and verified it using an *E. coli* gene expression compendium^15^. Previous studies^26,27^ reported binding sites of SoxS and MarA, responsible for oxidative stress mitigation^26^ and antibiotic resistance^28^ in the upstream region of the *cyuR* gene (Fig. 4A and Supplementary Data 1), implying its relevance to the stress resistance. Indeed, the expression of *cyuR* is highly correlated to the expression of 117 genes, mostly regulated by SoxS (the SoxS iModulon^15,29^, Fig. 4B and Supplementary Data 1). The expression level of *soxS* and *cyuR* was also highly correlated (Pearson correlation of 0.57, Fig. S4A), although the correlation between *cyuR* and *marA* was not high (Pearson correlation of 0.16, Fig. S4B). These observations confirm the CyuR regulation by SoxS.

We then further explored the regulation by CyuR in *E. coli* by revisiting a previously reported ChIP-exo dataset^10^ for CyuR and the iModulon analysis of the *E. coli* gene expression compendium^15^. So far, two binding sites have been reported. One binding site in the upstream of the *cyuPA* operon was thoroughly validated whereas the other one in the upstream of *cyuR* was yet to be confirmed (Fig. 4C and Supplementary Data 1). Interestingly, there are two genes, *mdlAB*, encoding putative transporters, co-localized with *cyuR* and also included in the SoxS iModulon, suggesting their co-expression. Indeed, the correlation between *cyuR* and *mdlA* was high (Pearson correlation of 0.71 for all 1035 samples or 0.77 for all samples except the *cyuR*-deleted strain, Fig. 4D, and Supplementary Data 1).

Since there was no predicted transcriptional start site in the intergenic region of *cyuR* and *mdlA*, it was inferred that they form an operon. The *mdlA* gene was up-regulated when *cyuR* was deleted and the oxidative-stress-inducing paraquat (PQ) was added (the YTF, Oxidative, ROS_TALE projects, see Supplementary Note 1 for details, Figs. 4E and S5, and Supplementary Data 1). Furthermore, the addition of several antibiotics up-regulated the expression of the *mdlA* gene in the Cation-Adjusted Mueller Hinton Broth (CAMHB) and the 90% Roswell Park Memorial Institute 1640 + 10% LB (RPMI + 10% LB) medium, but not in the M9 glucose medium. Its up-regulation was not observed when either *soxS* or *soxR* was deleted, supporting their regulatory roles for *mdlA* (and *cyuR*). In addition, since the deletion of *cyuR* up-regulated the expression of genes *mdlAB*, CyuR might be an auto-repressor. We also investigated the correlation between the expression of *cyuR* and *cyuP* (Fig. S6). Interestingly, the expression of these two genes was not highly correlated (Pearson correlation of 0.14). This is likely due to the absence of the effector of CyuR under the tested conditions. Cys was previously suggested to interact with CyuR, and essential for the CyuR-dependent activation of *cyuPA* operon by direct binding^8,9^. Collectively, these observations confirmed that CyuR is a Cys-responsive regulator for both *cyuR*-*mdlAB* and *cyuPA*.

### Identification of the binding motif of CyuR

**Fig. 5:**
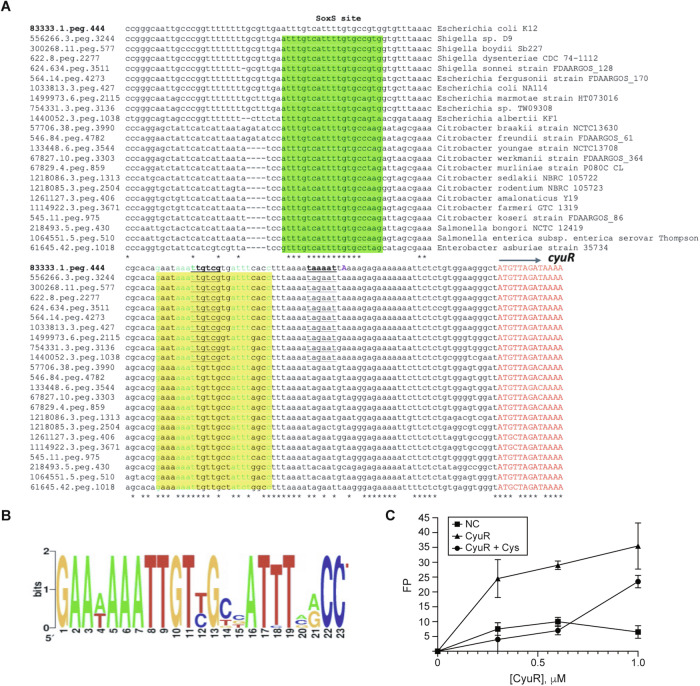
Sequence alignment of the upstream regions of CyuR in various microorganisms in the family of *Enterobacteriaceae.* **A** Sequence alignments of the upstream regions of *cyuR* in different microorganisms. Known SoxS binding motif and predicted CyuR-binding motif are colored in green and yellow, respectively. Asterisk indicates the commonality of corresponding bases. Each sequence starts with a feature ID that was given by the SEED database (https://www.theseed.org). **B** Sequence logos were generated based on the multiple alignments of CyuR-binding sites from multiple groups of *Enterobacteria* genomes (see Fig. S7) using the WebLogo tool (https://weblogo.berkeley.edu/logo.cgi). **C** The fluorescence polarization of CyuR and 10 nM fluorescently labeled 30 bp DNA fragment of the upstream sequence of the *cyuR* (see “Methods” section). This assay was performed in the presence and absence of 15 mM L-Cys with biological duplicate. *x*-axis and *y*-axis indicate CyuR concentration (μM) and fluorescence polarization, respectively. NC indicates the negative control which is HisR from *R. gnavus* (see “Methods” section).

We determined the binding motif of CyuR by investigating a consensus sequence^30^ and then validated the targets by employing in vitro fluorescence polarization assay. We hypothesized that the CyuR-binding site can be predicted from the alignment of the upstream regions of c*yuR*-*mdlAB* from diverse species, given it was found that *cyuR-mdlAB* is highly conserved in many Proteobacteria (Fig. S7); it is commonly present in *E. fergusonii*, *E. albertii, Shigella boydii, S. dysenteriae, S. sonei, Citrobacter freundii, C. youngae, C. koseri*, *C. farmeri, C. braakii, Salmonella bongori, S. enterica*, and *Enterobacter asburiae*. We aligned the *cyuR* upstream regions in 22 Enterobacteria, and identified a conserved 23-bp sequence, suggesting it is a putative binding motif for the regulator CyuR (Fig. 5A, yellow box). It is an imperfect palindrome with consensus GAAwAAATTGTxGxxATTTsyCC, where ‘x’ is either T or C, ‘s’ is either A or C, ‘y’ is either A or G, and ‘w’ is either A or T (Fig. 5B). This result was consistent with a previously reported binding position identified by ChIP-exo (Fig. 4C). In addition, the transcription of *cyuR* was predicted to be sigma 70 dependent^31^.

To confirm the CyuR-binding motif, we conducted a fluorescence polarization assay^32^ in the absence and presence of Cys (Fig. 5C, Supplementary Data 1, see “Methods” section). Purified CyuR was incubated at different concentrations of Cys (0, 0.3, 0.6, and 1.0 μM) supplemented with a 10 nM fluorescently labeled 30 bp DNA fragment that mimics the upstream sequence of the *cyuR* gene and negative control 30 bp DNA fragment. Notably, after a 1 h incubation, we observed that the FP signal increased as the amount of CyuR increased for the predicted DNA-binding fragment, and Cys is the effector for the CyuR-binding, but no increase in FP signal was detected for the negative control. This observation confirmed that the effector of CyuR is Cys and CyuR indeed binds the predicted consensus sequence.

### MdlA, which is co-expressed with CyuR, is a putative efflux pump for antibiotics

We hypothesized that MdlA or MdlB could function as an antibiotic resistance efflux pump because *mdlAB* were predicted to encode membrane proteins with a nucleotide-binding domain. This domain is highly conserved in ATP-binding cassette antibiotic efflux pumps^33^. Previously, a heterologous expression of SmdA, a homologous transporter to MdlA (an identity of 79%), from *Serratia marcescens* in *E. coli*, increased resistance against norfloxacin, tetracycline, and 4,6-diamidino-2-phenylindole^34^. This hypothesis was further supported by the fact that up-regulation of *mdlA* expression was observed in the presence of trimethoprim with sulfamethoxazole, ciprofloxacin, and ceftriaxone in the RPMI + 10% LB medium (Fig. 4E and Supplementary Data 1); the RPMI medium contains Cys.

**Fig. 6:**
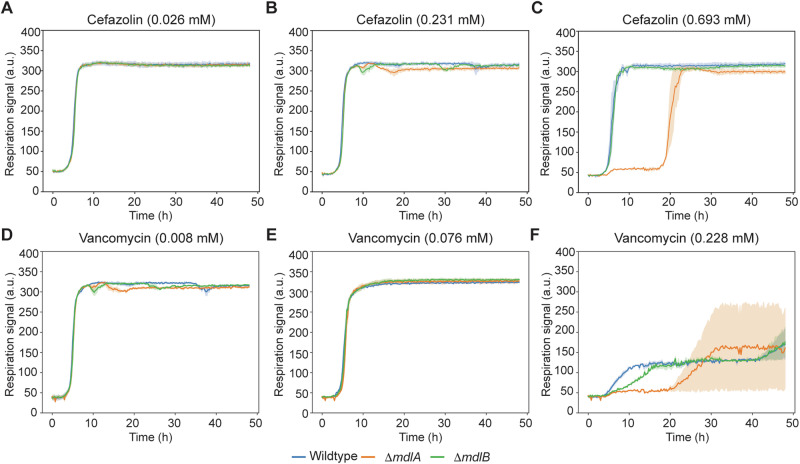
Phenotype microarray data for BW25113, BW25113 Δ*mdlA*, and BW25113 Δ*mdlB* in the presence of three different concentrations of cefazolin and vancomycin. **A**–**F** Time-course signals of BW25113 (wildtype, blue line), BW25113 Δ*mdlA* (orange line), and BW25113 Δ*mdlB* (green line) grown in a Biolog plate at three different concentrations of **A**–**C** cefazolin and **D**–**F** vancomycin. *x*-axis and *y*-axis indicate time (h) and respiration signal (a.u.), respectively. The shadow indicates the respiration signals of the two biological replicates.

We investigated the role of MdlA and MdlB using phenotype microarray plates. To test whether MdlA or MdlB can export antibiotics, we took BW25113, BW25113 Δ*mdlA*, and BW25113 Δ*mdlB* strains from the Keio collection (Table S1) and compared their phenotypes (Fig. 6A–F and Supplementary Data 1) using 11 C and 12B plates under the IF10b medium plus the presence of Cys. Notably, we found a difference in respiration signals from these strains in the presence of two antibiotics, cefazolin, and vancomycin. Although the three strains did not show large differences at the two low concentrations, the BW25113 Δ*mdlA* strain displayed a much longer lag period before growth at the highest concentrations, suggesting that MdlA is important for their efflux. Inconsistent with the previous study with *S. marcescens*^34^, no huge growth difference was observed with different tested levels of tetracycline (Fig. S8). Interestingly, the deletion of *mdlB* did not affect the viability in the presence of cefazolin. Although SmdA and SmdB in *S. marcescens* were suggested to form a heterodimer transporter^34^, it is likely that MdlA and MdlB are independent of each other or MdlA can be solely active.

Interestingly, when we complemented *mdlA* in the BW25113 Δ*mdlA* strain, the increased resistance to cefazolin and vancomycin was not detected (Table S8). Although the reason is not clear, the sub-optimal expression may affect the resistance; the overexpression of membrane transporter genes is sometimes problematic^35,36^. Nevertheless, these results suggest that *mdlA*, in the *cyuR-mdlAB* operon, encodes an efflux pump that has an effect for antibiotics, at least for cefazolin and vancomycin.

### Motif scanning suggests additional target genes of CyuR

We further explored the possibility that CyuR regulates additional target genes by motif scanning of the genome of *E. coli* MG1655 and comparative gene expression analysis. From this analysis, additional putative binding sites were identified in the upstream region of 25 genes (Table 1). Considering that the motif was identified from the upstream of *cyuR*-*mdlAB*, they were likely the targets for CyuR in the absence of Cys supplement (Fig. 4). For these genes, we revisited individual gene expression changes by the deletion of *cyuR*^10^ or supplementation of Cys. Notably, the expression levels of three genes, *setB* encoding sugar export transporter, *malE,* and *malK* encoding maltose ABC transporter, were commonly up-regulated in the two conditions. Considering their gene expression patterns, they can be repression targets by CyuR in the absence of Cys. Additionally, several genes were up-regulated by the supplementation of Cys. For example, *yaiV* (also called *iprA*), a DNA-binding regulator gene involved in resistance to oxidative stress^37^, *etp* encoding a lipid A modification gene, and *lapA* encoding a lipopolysaccharide assembly encoding gene were up-regulated, suggesting that these genes are potentially regulated by CyuR. The results were confirmed by RT-qPCR (Fig. S9). It should be noted that there were genes whose expression unexpectedly changed or even did not greatly change in both conditions. These observations can be understood as false positives or the presence of regulation by multiple transcription factors. Nevertheless, these results imply that CyuR may have further expanded regulatory roles for diverse metabolism in *E. coli*, which requires further detailed studies.

**Table 1:** Potential regulation targets of CyuR, suggested by motif scanning

#	Gene	Locus tag	Gene function	Position	Z-score	CyuR predicted DNA-binding site	Log_2_ Fold change	
							Δ*cyuR*^a^	Cys^b^
-	*cyuR*	b0447	DNA-binding transcriptional activator	−69	5.84	GAATAAATTGTcGtgATTTcaCC	n.a.	-
1	*yaiV*	b0375	Putative DNA-binding transcriptional factor	−75	5.41	GAAgAAATacTgGtAATTTAaTC	-	4.5
2	*etp*	b0982	Phosphotyrosine-protein phosphatase	−199	4.88	GGcaAAATcGCcAatATTTATCa	-	1.5
3	*ycfT*	b1115	Inner membrane protein (biofilm-related)	−143	4.8	GGcTAAATTGCcAtgtTTTATCa	-	7.9
4	*lapA*	b1279	Lipopolysaccharide assembly protein A	−50	4.69	GGtaAgtTgaCcAtAATTTATTC	-	1.4
5	*puuD*	b1298	Putrescine utilization	−42	4.84	GcAgtAATgGCgAtAATTTAgTC	−2.1	2.4
6	*pspF*	b1303	Psp membrane-stress response, σ54-enhancer binding protein	−179	4.88	GAAaAAATacCcAtAATgTtgTC	-	-
7	*pspA*	b1304	Psp membrane-stress response	−10	4.88	GAcaAcATTaTgGgtATTTtTTC	-	−2.9
8	*dbpA*	b1343	RNA helicase	−10	4.71	GAATAgATTGTgACcgcTTtTTC	-	-
9	*ydcP*	b1435	23S rRNA 5-hydroxycytidine C2501 synthase	−40	4.95	GctaAAATaGCcGCcATTTtTCa	-	-
10	*yoaF*	b1793	Lipoprotein	−160	4.92	GGgTAAATgcTgAttATTTAaTC	-	-
11	*dgcP*	b1794	Diguanylate cyclase	−45	4.92	GAtTAAATaaTcAgcATTTAcCC	-	−1.4
12	*zwf*	b1852	Glucose-6-P dehydrogenase	−233	4.61	GAtaAAAaaGTTGttATTTtTTt	-	−2.8
13	*yebK*	b1853	DNA-binding transcriptional repressor	−127	4.61	aAAaAAATaaCAACttTTTtaTC	-	-
14	*fruB*	b2169	Fructose-specific PTS	−192	4.58	GGAaAAATgGCAAaAAaTTgTgC	1.1	-
15	*setB*	b2170	Sugar export transporter	−198	4.58	GcAcAAtTTtTTGCcATTTtTCC	1.4	1.4
16	*yfaL*	b2233	Putative autotransporter adhesin	−277	4.79	GGcgAAATgGTTGtttTcTATTC	-	2.4
17	*raiA*	b2597	Ribosome-associated inhibitor A	−89	4.89	GAcaAAATTaTgAgAtTTTcaTC	2.2	−4.0
18	*araE*	b2841	Arabinose symporter	−5	4.7	GAAaAAATgGTTACtATcaATaC	-	1.6
19	*yhiJ*	b3488	DUF4049 protein	−49	4.97	GAtaAAATgtTAACtATgTATTC	-	3.4
20	*aldB*	b3588	Aldehyde dehydrogenase	−199	5.61	GAAgAAATTGTgGCgATTTATCg	1.2	-
21	*pfkA*	b3916	6-Phosphofructokinase	−148	4.77	GtATAAAaTaCcGCcATTTggCC	2.2	−3.7
22	*argC*	b3958	Arginine biosynthesis	−182	4.76	cGATAAATgGCgGtAATTTgTTt	-	−1.9
23	*malE*	b4034	Maltose ABC transporter	−178	4.93	GcAaAAATcGTgGCgATTTtaTg	1.4	1.8
24	*malK*	b4035	Maltose ABC transporter	−209	4.93	cAtaAAATcGCcACgATTTtTgC	2.7	3.9
25	*prfC*	b4375	Peptide chain release factor 3	−71	5.53	GGtaAAATaGCcGCAATTTtTCg	−1.1	−1.0

^a^Fold changes in gene expression when *cyuR* was deleted in *E. coli* MG1655^10^.

^b^Fold changes in gene expression by supplementing 5 mM Cys in *E. coli* W. Both studies were performed by using the same M9 medium. ‘-’ indicates a gene was not differentially expressed. ‘n.a.’ indicates not applicable. The underlined genes are potential regulation targets with strong evidence with the gene expression changes.

## Discussion

In this study, we investigated the effects of Cys supplementation to AMR and its related regulatory network in *E. coli* by utilizing a phenotype microarray assay and an iModulon activity analysis of gene expression profiles. We found that Cys supplementation led to the generation of H_2_S and subsequently increased resistance to many antibiotics. The iModulon analysis unveiled that Cys supplementation globally affects the transcriptome. Most importantly, it was observed that Cys supplementation noticeably relieved growth inhibition which was shown as decreased activities of stress response iModulons such as RpoS, GadXW, and RpoH. Interestingly, iModulons with increased activities are related to AA metabolism, translation and cell components, and carbon metabolism. These iModulons are essential for synthesizing cellular building blocks. Cys may also have an effect as a bacterial growth stimulator.

Another key finding was the regulatory roles of CyuR for Cys metabolism. We showed that CyuR is required for Cys-dependent resistance for at least nine antibiotics as test cases: demeclocycline, L-aspartic-β-hydroxamate, lincomycin, nalidixic acid, novobiocin, sulfadiazine, sulfamethazine, sulfamethoxazole, and sulfathiazole (Fig. 3). Furthermore, we also identified that CyuR is an auto-repressor that binds to a palindromic conserved motif “GAAwAAATTGTxGxxATTTsyCC” by comparative genomics and verified by a fluorescent polarization assay. Furthermore, it was shown that *mdlAB*, encoding an efflux pump, likely forms an operon together with *cyuR* which additionally highlights the close association of Cys metabolism with AMR. The motif was found in the upstream sequences of 25 genes, indicating that CyuR may have more expanded regulatory roles in diverse cellular metabolism. Some of these genes were indeed differentially expressed when *cyuR* was deleted or Cys was supplemented. In particular, they include genes encoding less-characterized transcription factors (e.g., YaiV and YebK) and membrane-related proteins (e.g., Etp, YcfT, PspA). Given our study suggested their associations to Cys metabolism in *E. coli*, future studies can be designed to investigate their roles and help to understand the full regulatory mechanisms of CyuR.

Currently, two opposite effects of Cys on microbial growth have been reported. Although bacteria can increase resistance to growth inhibitors by utilizing Cys, a high level of Cys can be toxic to bacteria^12,38,39^. This toxic effect can be higher in an anoxic condition where Cys is more stable than in an oxic environment. Several previous studies suggested Cys-induced growth inhibition mechanisms such as disruption of threonine deaminase activity^39^ and induction of amino acid starvation response^39^. This potential adverse effect has suggested as reasons for the less common presence of specific importers for Cys in bacteria^38^. Nevertheless, the utilization of Cys and subsequent generation of H_2_S is critical for antibiotic resistance. Interestingly, this beneficial effect varied depending on the types of antibiotics, which requires further studies with consideration of their mechanisms of inhibitions. Given that Cys is widely utilized as a food supplement and it increases antimicrobial resistance for *E. coli*, our study suggests that its potential harmful effect of Cys should be studied when treating bacterial infection diseases.

## Methods

### Bacterial strains and growth conditions

A list of the strains used in this study is listed in Table S1. MG1655 Δ*cyuR* was generated in a previous study^10^. Cultures were performed in an M9 medium (4 g L^−1^ glucose, 47.9 mM Na_2_HPO_4_, 22 mM KH_2_PO_4_, 0.5 g L^−1^ NaCl, 1 g L^−1^ NH_4_Cl, 2 mM MgSO_4_, 0.1 mM CaCl_2_, 250 μL L^−1^ Sauer Trace elements solution) except for cultures performed by using a phenotype microarray plate. Cells were initially grown overnight in the LB medium, and the cells were refreshed for 3 h in the same fresh medium. These cultures were washed and inoculated again into a fresh M9 medium in the presence or absence of L-Cys. Finally, the preculture was diluted back to OD_600_ 0.05 into a main culture medium. It should be noted that main cultures were performed in a microaerobic condition (i.e., no shaking) to avoid detrimental effects of aeration to Cys-related metabolism^9^.

### Phenotype microarray

*E. coli* strains were phenotyped by using microarray plates and an Omnilog system purchased from Biolog (https://www.biolog.com, Hayward, CA). These strains were inoculated from a corresponding glycerol stock to an LB medium. The next day, the cultures were re-inoculated in an IF10b medium (Biolog) and further incubated overnight. Then, the cultures were washed, diluted at the recommended OD_600_ (approximately 0.08) in the fresh IF10b or IF10a (CyuR complementation) medium, and incubated in the 11 C or 12B microplates containing diverse antibiotics at four different concentrations (Tables S3 and S4) at 37 °C. Bacterial respiration was monitored by using Biolog-redox dye with or without supplementation of 5 mM Cys with two biological replicates. For the CyuR complementation, MG1655 Δ*cyuR cyuR* was generated by introducing the pCA24N-*cyuR* plasmid^9^ into the *cyuR*-deletion strain. 34 μg/mL and 0.2 mM of chloramphenicol and isopropyl β-D-1-thiogalactopyranoside (IPTG) were included to maintain the plasmid and to express *cyuR* under the P_T5- lac_ promoter^9^. For the MdlA complementation, the pCA24N-*mdlA* plasmid^9^ and NC was introduced to the *mdlA*-deletion mutant and cultivated identically to the *cyuR* complementation assay. *mdlA* was also expressed under the same P_T5- lac_ promoter. Chloramphenicol was included in only pre-cultures to avoid deleterious overexpression of the transcription factor and the membrane protein MdlA.

### Transcriptome sequencing (RNA-Seq) and iModulon activity analysis

For transcriptome sequencing, total RNA was isolated from *E. coli* W grown in the M9 medium as described above. Pre-cultures were prepared in the LB medium and the overnight cultured cells were washed twice with the M9 medium and inoculated at an OD_600_ of 0.05 in the same medium. To induce redox-relative stress, we included 15 μg mL^−1^ of ampicillin^2^. 5 mM Cys was added as a treatment. The cells were collected at an OD_600_ of 0.6 and treated using the Qiagen RNA-protect reagent (Hilden, Germany) according to the instruction from the manufacturer. Pelleted cells were stored at −80 °C, and after cell resuspension and partial lysis, they were ruptured with a beat beater. Subsequently, the total RNA was extracted using a Quick-RNA Fungal/Bacterial Microprep kit from Zymo Research (Irvine, CA, USA) by following the manufacturer’s protocol. After total RNA extraction, ribosomal RNA was removed^40^ and the quality was assessed using an Agilent Bioanalyser using an RNA 6000 kit. Paired-end RNA sequencing libraries were prepared by using a KAPA RNA HyperPrep kit from Kapa Biosystems (Wilmington, MA, USA) as described previously^41^. Raw-sequencing reads were aligned to the reference genome of the *E. coli* W strain (accession number: CP002185.1) using Bowtie2^42^. Transcripts per million calculation and differentially expressed gene analysis were performed by DESeq2 (v1.22.1)^43^. Transcriptome sequencing results were validated by performing RT-qPCR for *yaiV* and *ept* with using *rpoA* as a control. 1 μg of total RNA was converted to cDNA using SuperScript III Reverse Transcriptase and random hexamer primers. qPCR was run in a BioRad CFX connect thermal cycler by using primer pairs (Table S9) designed for the seven genes and one housekeeping gene (*rpoA*) and GoTaq G2 Polymerase from Promega (Madison, Wisconsin, USA). iModulon activity analysis was performed by using the Pymodulon package (https://github.com/SBRG/pymodulon)^14^.

### Prediction of the binding site of CyuR and motif scanning

The potential CyuR-binding site was identified by a comparative genomics approach using multiple sequence alignments. Orthologs of the *E. coli cyuR* gene in other Proteobacteria, as well as multiple sequence alignments of the orthologous upstream region, were identified using the PubSEED comparative genomics platform^44^. CyuR-binding site logos were obtained by using the WebLogo tool^45^. The aligned CyuR sites from *E. coli* and other proteobacteria were used as a training set to build a Positional Weight Matrix (PWM) Positional nucleotide weights in the CyuR-PWM and Z-scores of candidate sites were calculated as the sum of the respective positional nucleotide weights.

Genome scanning for identifying additional candidate CyuR-binding sites was performed using the Genome Explorer software^46^. The threshold for the site search was defined as the lowest score observed in the training set (Z-score = 4.5).

### In vitro DNA-binding assay for CyuR

A single colony of the *cyuR* overexpressing strain of *E. coli* obtained from the ASKA collection^47^, was inoculated into a LB medium from an agar plate containing chloramphenicol and grown at 37 °C. After overnight, the culture was further passaged to a 50 mL fresh medium. After OD_600_ reached 0.8, 0.8 mM isopropyl β-D-1-thiogalactopyranoside was added. The cultures were incubated at 24 °C overnight with continuous shaking, and cells were collected by centrifugation and lysed as previously described^48^. The CyuR recombinant protein containing an N-terminal 6хHis tag was purified by Ni-chelation chromatography from the soluble fraction as described^48,49^. The insoluble fraction was solubilized in 7 M urea At-buffer and purified on a Ni-NTA mini-column (Qiagen Inc.) with At-buffer (100 mM Tris-HCl buffer), pH 8, 0.5 mM NaCl, 5 mM imidazole, and 0.3% Brij^TM^, β-mercaptoethanol with 7 M urea. The expression of the proper size of CyuR was confirmed by performing an SDS-PAGE analysis.

The interaction of the purified CyuR protein with its specific DNA-binding site was assessed using fluorescence polarization with fluorescently labeled 28-bp orthologous DNA fragment from *Citrobacter braakii* (5′-aggAAAATTGTTGCCATTTAGCCTTTAAAATgga), containing the predicted orthologous CyuR-binding site and flanked on each side by extra guanine residues. For each DNA fragment, two complementary single-stranded oligonucleotides were synthesized by Integrated DNA Technologies (IDT), at which the fragments were labeled by 6-carboxyfluorescein at 5′ end. The double-stranded DNA fragments were obtained by annealing the labeled oligonucleotides with unlabeled complementary oligonucleotides at a 1:10 ratio. The DNA fragment for HisR from *R. gnavus* was utilized as CyuR DNA-binding negative control. The negative control is 20-bp binding site upstream of the *hisX* gene (Rumgna_00302), CAGTTTAGTATAGTAAAGT, that was linked to the GGGGG sequences at both ends to improve annealing of single-stranded DNA fragments. The single-stranded labeled and unlabeled DNA oligos were synthesized by IDT and double-stranded DNA obtained as described^50^. The obtained fluorescently labeled DNA fragments (10 nM) were incubated for 1 h at 30 °C with increasing concentrations of recombinant CyuR protein in the assay mixture (0.1 mL) in 96-well black plates. The binding buffer contained 20 mM Tris-HCl (pH 7.5), 0.1 M NaCl, 0.5 mM EDTA, 10 mM MgSO_4_, 2 mM DTT, and 5 μg mL^−1^ herring sperm DNA. The fluorescence-labeled DNA was detected by using a Beckman Coulter DTX 880 Multimode Detector. We monitored the fluorescent polarization (FP) in the absence or presence of Cys (15 mM).

### Statistics and reproducibility

*T*-testing was performed for groups that have three or more samples. Unless otherwise stated, all experiments were performed in biological duplicates for reproducibility.

## Supplementary information

Supplementary Information

Description of Additional Supplementary File

Supplementary Data 1

reporting-summary

## Data Availability

The RNA-seq raw data files were uploaded to Gene Expression Omnibus (https://www.ncbi.nlm.nih.gov/geo) under GSE215167 as an accession ID. Source data is attached to the manuscript. All other data are available from the corresponding authors on reasonable request.
