# Supplementary material for: CyuR is a dual regulator for L-cysteine dependent antimicrobial resistance in Escherichia coli
Source: Commun Biol. 2024 Sep 17;7:1160. doi: 10.1038/s42003-024-06831-0 (PMC11408624; doi:10.1038/s42003-024-06831-0)
Supplement: Supplementary file 1 — Supplementary Information [file 42003_2024_6831_MOESM1_ESM.pdf]

Supplementary Information for

CyuR is a Dual Regulator for L-Cysteine Dependent Antimicrobial Resistance in *Escherichia coli*

Irina A. Rodionova[1,a,*], Hyun Gyu Lim[1,2,a], Ye Gao[1,4], Dmitry A Rodionov[3], Ying Hutchison[1], Richard Szubin[1], Christopher Dalldorf[1], Jonathan Monk[1], Bernhard O. Palsson[1,5,6,*]

[1]Department of Bioengineering, Division of Engineering, University of California San Diego, La Jolla, CA 92093, USA.

[2]Department of Biological Sciences and Bioengineering, Inha University, Incheon, Korea

[3]Sanford-Burnhams-Prebys Medical Discovery Institute, La Jolla, CA 92093, USA.

[4]The Second Hospital of Shandong University, Jinan, Shandong 250033, P.R. China.

[5]Department of Pediatrics, University of California San Diego, La Jolla, CA 92093, USA

[6]Novo Nordisk Foundation Center for Biosustainability, Technical University of Denmark, Lyngby 2800, Denmark

[a] indicates equal contributions

*To whom correspondence should be addressed:

Dr. Irina A. Rodionova (irodionova@ucsd.edu)

Prof. Bernhard O. Palsson (palsson@ucsd.edu)

Supplementary Note

Supplementary Note 1. *mdlA* expression changes identified in PRECISE-1K

We investigated the expression correlation and *mdlA* PRECISE-1K contains a collection of 1,035 *E. coli* gene expression profiles, collected in diverse environmental conditions from a total of 45 projects[1,2] (**Figure S1**). Project names were given in the previous large-scale transcriptome analysis studies. We observed changed expression levels of *mdlA* in samples in four projects: yTF, Oxidative, ROS_TALE, Abx_media, which corresponds to Ye et al., 2018[3], Seo et al., 2015[4], Rychel et al., 2022[5], Sastry et al., 2021[6].

1) The yTF project[3] investigated transcriptome changes in *E. coli* with a single deletion of either *yafC*, *cyuR*, *ybiH*, *ydcI*, *yddM*, *yeiE*, *yheO*, *yiaJ*, or *yieP*. *mdlA* expression was increased in a *cyuR* deleted *E. coli* K-12 MG1655 strain. This observation indicates CyuR negatively regulates *mdlA* expression.
2) The Oxidative project[4] investigated the role of OxyR, SoxS, and SoxR, which are important for mitigating oxidative stress in *E. coli*. The increased expression of *mdlA* by the addition of 250 µM paraquat was not observed in *soxR* and *soxS* deleted *E. coli* strains, implying potential regulation by the SoxSR system for *mdlA* expression. The fold change was calculated based on WT without PQ (Log_2 FC = 0).
3) The ROS_TALE project[5] investigated transcriptome changes in the wildtype and PQ-tolerized strains via adaptive laboratory evolution[6]. As observed in the samples from the Oxidative project, increased expression of *mdlA* was observed in PQ added conditions regardless of strains.
4) The Abx_media project[6] investigated transcriptome changes in *E. coli* depending on types of antibiotics in three different media. *mdlA* expression was affected by several antibiotics in CAMHB or RPMI+10% LB medium.

Supplementary Tables

Supplementary Table 1. Stains used in this study

Name	Genotype	Reference
MG1655	*E. coli* K-12 MG1655 wildtype	[3]
MG1655 Δ*cyuR*	*E. coli* K-12 MG1655 Δ*cyuR*	[3]
MG1655 Δ*cyuR cyuR*	*E. coli* K-12 MG1655 Δ*cyuR* {pCA24N-*cyuR*}	This study
BW25113	Keio collection	[7]
BW25113 Δ*mdlA*	Keio collection	[7]
BW25113 Δ*mdlB*	Keio collection	[7]
BW25113 Δ*mdlA mdlA*	Keio collection, {pCA24N- *mdlA*}	This study
W	*E. coli* W	ATCC 9637
GN02094	Clinical *E. coli* isolate 1,<br>Biosample id: SAMN03922915	[8]
GN02007	Clinical *E. coli* isolate 2,<br>Biosample id: SAMN03922907	[8]
GN02148	Clinical *E. coli* isolate 3,<br>Biosample id: SAMN03922919	[8]
BL21(DE3) *cyuR*	*E. coli* BL21(DE3) {pCA24N-*cyuR*}	[9]

Supplementary Table 2. Phylogroups of model *E. coli* strains and clinical isolates

Strains	Phylogroups
E. coli K-12 MG1655	A
E. coli BL21	A
E. coli BW25113	A
E. coli W	B1
GN02148	B2
GN02094	D
GN02007	B2

Supplementary Table 3. Concentrations of antibiotics in Biolog plate PM11C

Biolog plates are commercially available plates from Biolog for testing antibiotic resistance in a high-throughput manner. Phenotype Microarray (PM) 11C plate contains four different levels of diverse growth inhibitors. The concentrations are given below.

Well	Substrate	mM
A1	Amikacin	0.004
A2	Amikacin	0.012
A3	Amikacin	0.037
A4	Amikacin	0.11
A5	Chlortetracycline	0.002
A6	Chlortetracycline	0.005
A7	Chlortetracycline	0.016
A8	Chlortetracycline	0.048
A9	Lincomycin	0.055
A10	Lincomycin	0.165
A11	Lincomycin	0.495
A12	Lincomycin	1.484
B1	Amoxicillin	0.001
B2	Amoxicillin	0.003
B3	Amoxicillin	0.01
B4	Amoxicillin	0.03
B5	Cloxacillin	0.087
B6	Cloxacillin	0.262
B7	Cloxacillin	0.787
B8	Cloxacillin	2.36
B9	Lomefloxacin	0.005
B10	Lomefloxacin	0.014
B11	Lomefloxacin	0.043
B12	Lomefloxacin	0.129
C1	Bleomycin	0.003
C2	Bleomycin	0.01
C3	Bleomycin	0.031
C4	Bleomycin	0.092
C5	Colistin	0.005
C6	Colistin	0.014
C7	Colistin	0.042
C8	Colistin	0.126
C9	Minocycline	0.002
C10	Minocycline	0.005
C11	Minocycline	0.016
C12	Minocycline	0.049
D1	Capreomycin	0.002
D2	Capreomycin	0.005
D3	Capreomycin	0.015
D4	Capreomycin	0.046
D5	Demeclocycline	0.001
D6	Demeclocycline	0.002
D7	Demeclocycline	0.007
D8	Demeclocycline	0.021
D9	Nafcillin	0.104
D10	Nafcillin	0.313
D11	Nafcillin	0.939
D12	Nafcillin	2.816

Well	Substrate	mM
E1	Cefazolin	0.026
E2	Cefazolin	0.077
E3	Cefazolin	0.231
E4	Cefazolin	0.693
E5	Enoxacin	0.006
E6	Enoxacin	0.019
E7	Enoxacin	0.058
E8	Enoxacin	0.175
E9	Nalidixic acid	0.003
E10	Nalidixic acid	0.009
E11	Nalidixic acid	0.028
E12	Nalidixic acid	0.085
F1	Chloramphenicol	0.002
F2	Chloramphenicol	0.006
F3	Chloramphenicol	0.019
F4	Chloramphenicol	0.058
F5	Erythromycin	0.004
F6	Erythromycin	0.011
F7	Erythromycin	0.033
F8	Erythromycin	0.098
F9	Neomycin	0.004
F10	Neomycin	0.011
F11	Neomycin	0.033
F12	Neomycin	0.099
G1	Ceftriaxone	0.001
G2	Ceftriaxone	0.004
G3	Ceftriaxone	0.012
G4	Ceftriaxone	0.037
G5	Gentamicin	0.002
G6	Gentamicin	0.005
G7	Gentamicin	0.016
G8	Gentamicin	0.049
G9	Potassium tellurite	0.008
G10	Potassium tellurite	0.024
G11	Potassium tellurite	0.071
G12	Potassium tellurite	0.213
H1	Cephalothin	0.002
H2	Cephalothin	0.005
H3	Cephalothin	0.016
H4	Cephalothin	0.049
H5	Kenamycin	0.004
H6	Kenamycin	0.011
H7	Kenamycin	0.032
H8	Kenamycin	0.096
H9	Ofloxacin	0.002
H10	Ofloxacin	0.006
H11	Ofloxacin	0.018
H12	Ofloxacin	0.055

Supplementary Table 4. Concentrations of antibiotics in Biolog plate PM12B

PM12B plate contains four different levels of diverse growth inhibitors. The concentrations are given below.

Well	Substrate	mM
A1	Penicillin G	0.01
A2	Penicillin G	0.029
A3	Penicillin G	0.086
A4	Penicillin G	0.259
A5	Tetracycline	0.001
A6	Tetracycline	0.002
A7	Tetracycline	0.006
A8	Tetracycline	0.017
A9	Carbenicillin	0.004
A10	Carbenicillin	0.013
A11	Carbenicillin	0.039
A12	Carbenicillin	0.116
B1	Oxacillin	0.112
B2	Oxacillin	0.337
B3	Oxacillin	1.011
B4	Oxacillin	3.034
B5	Penimepicycline	0.002
B6	Penimepicycline	0.005
B7	Penimepicycline	0.016
B8	Penimepicycline	0.049
B9	Polymyxin B	0.00004
B10	Polymyxin B	0.0011
B11	Polymyxin B	0.00034
B12	Polymyxin B	0.00103
C1	Paromomycin	0.0002
C2	Paromomycin	0.0005
C3	Paromomycin	0.0016
C4	Paromomycin	0.0049
C5	Vancomycin	0.008
C6	Vancomycin	0.025
C7	Vancomycin	0.076
C8	Vancomycin	0.228
C9	DL-Serine hydroxamate	0.312
C10	DL-Serine hydroxamate	0.937
C11	DL-Serine hydroxamate	2.811
C12	DL-Serine hydroxamate	8.434
D1	Sisomicin	0.00003
D2	Sisomicin	0.0001
D3	Sisomicin	0.00089
D4	Sisomicin	0.00088
D5	Sulfamethazine	0.007
D6	Sulfamethazine	0.021
D7	Sulfamethazine	0.62
D8	Sulfamethazine	0.187
D9	Novobiocin	0.078
D10	Novobiocin	0.233
D11	Novobiocin	0.698
D12	Novobiocin	2.093

Well	Substrate	mM
E1	2,4-Diamino-6,7-Diisopropylpteridine	0.008
E2	2,4-Diamino-6,7-Diisopropylpteridine	0.023
E3	2,4-Diamino-6,7-Diisopropylpteridine	0.068
E4	2,4-Diamino-6,7-Diisopropylpteridine	0.203
E5	Sulfadiazine	0.004
E6	Sulfadiazine	0.012
E7	Sulfadiazine	0.037
E8	Sulfadiazine	0.112
E9	Benzethonium chloride	0.01
E10	Benzethonium chloride	0.031
E11	Benzethonium chloride	0.092
E12	Benzethonium chloride	0.276
F1	Tobramycin	0.00006
F2	Tobramycin	0.00019
F3	Tobramycin	0.00056
F4	Tobramycin	0.00169
F5	Sulfathiazole	0.004
F6	Sulfathiazole	0.011
F7	Sulfathiazole	0.034
F8	Sulfathiazole	0.102
F9	5-Fluoroorotic acid	0.031
F10	5-Fluoroorotic acid	0.093
F11	5-Fluoroorotic acid	0.278
F12	5-Fluoroorotic acid	0.833
G1	Spectinomycin	0.003
G2	Spectinomycin	0.01
G3	Spectinomycin	0.029
G4	Spectinomycin	0.088
G5	Sulfamethoxazole	0.002
G6	Sulfamethoxazole	0.005
G7	Sulfamethoxazole	0.014
G8	Sulfamethoxazole	0.043
G9	L-Aspartic-b-hydroxamate	0.087
G10	L-Aspartic-b-hydroxamate	0.261
G11	L-Aspartic-b-hydroxamate	0.783
G12	L-Aspartic-b-hydroxamate	2.349
H1	Spiramycin	0.052
H2	Spiramycin	0.156
H3	Spiramycin	0.467
H4	Spiramycin	1.4
H5	Rifampicin	0.001
H6	Rifampicin	0.003
H7	Rifampicin	0.01
H8	Rifampicin	0.029
H9	Dodecyltrimethyl ammonium bromide	0.035
H10	Dodecyltrimethyl ammonium bromide	0.106
H11	Dodecyltrimethyl ammonium bromide	0.319
H12	Dodecyltrimethyl ammonium bromide	0.958

Supplementary Table 5. Effects of Cys supplementation to diverse growth inhibitors

	Inhibitor	Max conc. (mM)[a]	Increased respiration signal by Cys supplementation[b]					
			W	GN02007	GN02094	GN02148	MG1655	MG1655 Δ*cyuR*
1	Amikacin	0.11	↑	-	-	↑	-	-
2	Amoxicillin	0.03	↑	-	-	↑	-	-
3	Benzethonium Chloride	0.092	△	↑	↑	↑	△	△
4	Bleomycin	0.092	△	-	-	↑	-	-
5	Capreomycin	0.046	↑	-	-	↑	-	-
6	Carbenicillin	0.116	△	-	-	↑	↑	↑
7	Cefazolin	0.693	↑	-	-	↑	-	-
8	Ceftriaxone	0.037	↑	-	↑	↑	-	-
9	Cephalothin	0.049	↑	-	-	↑	-	-
10	Chloramphenicol	0.019	↑	↑	↑	↑	↑	↓
11	Chlortetracycline	0.016	-	↑	↑	↑	△	△
12	Cloxacillin	0.787	↑	↑	↑	↑	↑	↑
13	Colistin	0.014	↑	↑	↑	↑	△	△
14	Demeclocycline	0.007	↑	↑	↑	↑	↑	△
15	2,4-Diamino-6,7-diisopropylpteridine	0.068	△	△	-	↑	↑	↓
16	Dodecyltrimethylammonium bromide	0.319	△	↑	↑	↑	△	△
17	Enoxacin	0.058	↑	↑	↑	↑	△	△
18	Erythromycin	0.098	↑	↑	↑	↑	-	△
19	5-Fluoroorotic acid	0.833	△	△	△	△	-	-
20	Gentamicin	0.049	↑	-	-	-	-	-
21	Kanamycin	0.096	↑	-	-	↑	-	-
22	L-Aspartic-β-hydroxamate	2.349	↑	↑	↑	↑	↑	△
23	Lincomycin	1.484	↑	↑	↑	↑	↑	△

24	Lomefloxacin	0.129	↑	↑	↑	↑	-	△
25	Minocycline	0.016	↑	↑	↑	↑	△	△
26	Nafcillin	0.939	↑	↑	↑	↑	↑	↑
27	Nalidixic acid	0.028	↑	↓	↓	↓	↑	△
28	Neomycin	0.099	↑	-	-	↑	-	-
29	Novobiocin	0.233	↑	△	△	↑	↑	△
30	Ofloxacin	0.055	↑	↑	↑	↑	-	△
31	Oxacillin	1.011	↑	-	↑	↑	↑	↑
32	Paromomycin	0.0016	△	↑	↑	↑	↑	-
33	Penicillin G	0.259	↑	-	-	↑	↑	↑
34	Penimepicycline	0.049	↑	↑	↑	↑	-	↓
35	Polymyxin B	0.00034	-	↓	↓	↓	△	△
36	Potassium tellurite	0.213	↑	↑	↑	↑	-	↑
37	Rifampicin	0.029	-	↑	↑	↑	-	-
38	Sisomicin	0.00089	△	-	-	-	△	-
39	D,L-serine hydroxamate	8.434	↑	↑	↑	↑	△	△
40	Spectinomycin	0.088	↑	↑	↑	↑	-	-
41	Spiramycin	1.4	↑	↑	↑	↑	↑	↑
42	Sulfadiazine	0.112	↑	↑	↑	↑	↑	△
43	Sulfamethazine	0.187	↑	↑	↑	↑	↑	△
44	Sulfamethoxazole	0.043	△	↑	↑	↑	↑	△
45	Sulfathiazole	0.102	△	↑	△	↑	↑	△
46	Tetracycline	0.017	△	↑	-	↑	△	△
47	Tobramycin	0.00169	↑	↑	↑	↑	-	-
48	Vancomycin	0.228	↑	↑	↑	↑	-	↓

Supplementary Table 6. iModulon activity changes by the addition of 5 mM Cys

iModulon name	Explained Variances (%)	Activity changes	Functional category	Regulator
gcvB	2.8	25.3	Amino Acid Metabolism	gcvB
FlhDC-2	1.7	21.1	Extracellular Structures	FlhDC
SSW SNPs	1.8	20.7	ALE Effects	
nquinone ALE 3	1.5	20.0	Energy Metabolism	
FucR/AllR/AraC	1.2	18.7	Nitrogen Metabolism	FucR/AllR/AraC
FliA	0.9	16.5	Extracellular Structures	FliA
PlaR	0.8	16.1	Carbon Metabolism	PlaR+RpoE+Crp+IHF
Crp-1	0.8	15.6	Carbon Metabolism	Crp
Capsule	0.8	14.6	Extracellular Structures	RcsAB
YgeV	0.7	14.4	Carbon Metabolism	YgeV
YcjW	0.8	14.3	Carbon Metabolism	YcjW
PaaX	0.7	14.0	Carbon Metabolism	Crp+IHF+PaaX+SlyA
Oxidoreductase	0.6	13.8	Redox Stress	
Cytochrome c	0.5	13.5	Energy Metabolism	FlhDC+IHF+NsrR+NarL+NarP+Fis+Fnr
Fur-1	0.7	13.5	Metal Homeostasis	Fur
Tryptophanase	0.5	12.9	Amino Acid Metabolism	Crp+TorR+Nac+L-tryptophan
UC-9	0.6	12.5	Unknown	
Sulfoquinovose	0.4	12.1	Carbon Metabolism	CsqR
Phosphate-2	0.5	12.0	Phosphate Metabolism	PhoB
Rhamnose	0.5	11.9	Carbon Metabolism	Crp+RhaS
UC-7	0.5	11.4	Unknown	
FlhDC-1	0.4	10.9	Extracellular Structures	FlhDC
EvgA	0.5	10.8	Envelope Stress	EvgA
Gluconate	0.4	10.4	Carbon Metabolism	GntR+Crp
UC-3	0.3	10.0	Unknown	
Curli-1	0.4	10.0	Extracellular Structures	RpoS+FliZ+CpxR+CsgD+BtsR
pts ALE	0.3	10.0	Energy Metabolism	
Xylose	0.4	9.8	Carbon Metabolism	Crp+XylR
YgbI	0.3	9.7	Carbon Metabolism	YgbI
yneP	0.3	9.3	Unknown	
Thr/Ser-1	0.3	9.1	Amino Acid Metabolism	TdcA+TdcR+IHF+Fnr+Crp
Hot TALE 16	0.3	8.8	Unknown	
SCFA	0.4	8.3	Carbon Metabolism	RpoN+AtoC+IHF
Acetate	0.3	7.8	Carbon Metabolism	
Sorbitol	0.2	7.4	Carbon Metabolism	SrlR+GutM+Crp+H-NS
Crp-2	0.2	7.3	Carbon Metabolism	Crp

Quorum Sensing	0.2	7.1	Quorum Sensing	Crp+LsrR
Putrescine	0.1	7.0	Nitrogen Metabolism	RpoS+ArcA+PuuR
baeR KO	0.4	6.9	Envelope Stress	
Thr/Ser-2	0.2	6.9	Amino Acid Metabolism	Fnr+Crp+IHF+TdcA+TdcR
IS Elements-2	0.0	6.2	Unknown	
Sugar Diacid	0.1	6.1	Carbon Metabolism	CdaR
yafF	0.1	6.1	Unknown	
malE OE	0.2	6.0	Carbon Metabolism	
Efflux Pump	0.2	5.9	Resistance	
Salicylic Acid	0.1	5.9	Redox Stress	
Cellulose	0.2	5.9	Extracellular Structures	
Maltose	0.1	5.9	Carbon Metabolism	MalT
ypjJ	0.1	5.8	Unknown	
NrdR	0.1	5.7	Nucleotide Metabolism	NrdR
BasR	0.1	5.6	Extracellular Structures	BasR
Nitrogen	0.1	5.4	Nitrogen Metabolism	Nac
Nitrate/Nitrite	0.0	5.4	Nitrogen Metabolism	NarL+Fnr
yceO	0.1	5.4	Unknown	
Propionate	0.1	5.1	Carbon Metabolism	PrpR+RpoN+Cra+Crp
Flagella	0.1	5.1	Extracellular Structures	
NtrC-3	0.1	5.1	Nitrogen Metabolism	NtrC+RpoN
ROS TALE Del-2	0.1	-5.1	Unknown	
yqhI	0.1	-5.2	Unknown	
cyoB/ndh/nuoB KO-2	0.2	-5.5	Energy Metabolism	
ydfB	0.1	-5.8	Unknown	
cyoB/ndh/nuoB KO-1	0.2	-6.0	Energy Metabolism	
crp KO-1	0.1	-6.0	Carbon Metabolism	
PAL 26 Del	0.2	-6.1	Unknown	
tpiA KO	0.1	-6.2	Carbon Metabolism	
Leu/Val/Ile	0.1	-6.2	Amino Acid Metabolism	Lrp+IHF+Ile-tRNA+Leu-tRNA+Val-tRNA+ppGpp
N Reduction	0.2	-6.3	Energy Metabolism	FlhDC+ModE+NarP+NarL+Fnr
ymgI	0.0	-6.4	Unknown	
pgi KO	0.2	-6.7	Carbon Metabolism	
Nucleoside	0.1	-6.8	Nucleotide Metabolism	Crp+CytR
NDH-1	0.3	-7.0	Energy Metabolism	NarL+ArcA+IHF+Fnr+Fis+ryhB
pts KO	0.0	-7.0	Carbon Metabolism	
GadX	0.2	-7.2	Envelope Stress	RpoS+GadX
Thiamine-1	0.3	-7.3	Cofactor Metabolism	Thiamine diphosphate

Leucine	0.2	-8.0	Amino Acid Metabolism	LeuO+Leu-tRNA+ppGpp
Isc System	0.3	-8.2	Cofactor Metabolism	ryhB/IscR
Lysine/T2SS	0.2	-8.2	Amino Acid Metabolism	ArgP
Ferric Citrate	0.1	-8.2	Metal Homeostasis	PdhR+HypT+Nac+Crp+Fur+FecI
RpoE	0.3	-8.7	Temperature Shock	RpoE
Glutamine	0.4	-8.9	Nitrogen Metabolism	NtrC
IS5	0.0	-9.1	Unknown	
Glutarate	0.3	-9.1	Carbon Metabolism	RpoS+H-NS+Crp+Lrp+GlaR+ppGpp
Fnr-1	0.4	-9.3	Energy Metabolism	Fnr
Fnr-3	0.3	-9.3	Energy Metabolism	Fnr
minicoli KOs	0.1	-9.5	Genome Reduction	
Suf System	0.2	-9.5	Metal Homeostasis	NsrR+IHF+IscR+OxyR+Fur+ppGpp
DhaR	0.3	-9.8	Carbon Metabolism	DhaR
Arginine	0.3	-10.1	Amino Acid Metabolism	ArgR
ldrC	0.0	-10.4	Unknown	
Microaerobic	0.3	-10.5	Energy Metabolism	RpoS+Fis+NarP+NarL+AppY+YdeO+ArcA+IscR
Histidine	0.4	-10.5	Amino Acid Metabolism	His-tRNA+DksA
Phage Shock	0.4	-10.5	Phage Shock	RpoN+IHF+PspF
ymiB	0.0	-11.3	Unknown	
UC-4	0.7	-12.2	Unknown	
UC-2	0.9	-13.6	Unknown	
RpoH	0.8	-13.9	Temperature Shock	RpoH
Cysteine-1	0.8	-14.1	Amino Acid Metabolism	CysB
LPS	0.0	-14.8	Extracellular Structures	
Translation	1.6	-18.2	Translation	DksA
GadXW	1.4	-18.4	Envelope Stress	GadX+GadW
e14 Del	0.0	-21.6	Unknown	
RpoS	2.3	-21.8	Global Stress	RpoS
IS Elements-1	0.0	-31.4	Unknown	

Supplementary Table 7. Complementation assay of *cyuR* in a Biolog plate

Antibiotics	Concentration (mM)	Strains		
		MG1655	MG1655 Δ*cyuR cyuR*	MG1655 Δ*cyuR*
Novobiocin	0.078	+++	+++	++
Novobiocin	0.233	++	+++	+
Novobiocin	0.698	-	-	-
Novobiocin	2.093	-	-	-
2,4-Diamino-6,7-Diisopropylpteridine	0.008	+++	+++	++
2,4-Diamino-6,7-Diisopropylpteridine	0.023	++	++	-
2,4-Diamino-6,7-Diisopropylpteridine	0.068	+	-	-
2,4-Diamino-6,7-Diisopropylpteridine	0.203	-	-	-
Sulfadiazine	0.004	+++	+++	+++
Sulfadiazine	0.012	+++	+++	+
Sulfadiazine	0.037	++	+	+
Sulfadiazine	0.112	++	+	+
Sulfamethoxazole	0.002	+++	+++	+++
Sulfamethoxazole	0.005	++	+++	++
Sulfamethoxazole	0.014	++	+	+
Sulfamethoxazole	0.043	++	+	+
L-aspartic-b-hydroxamate	0.087	+++	+++	+++
L-aspartic-b-hydroxamate	0.261	+++	+++	++
L-aspartic-b-hydroxamate	0.783	++	++	+
L-aspartic-b-hydroxamate	2.349	++	++	+

The wildtype (MG1655), the *cyuR* mutant (MG1655 **Δ***cyuR*) and the *cyuR*-complemented strain (MG1655 **Δ***cyuR cyuR*) were grown in a Biolog PM12B plate to validate the role of CyuR in the increased resistance. We observed improved viability with the complementation of *cyuR* in the presence of novobiocin, 2,4-diamino-6,7-diisopropylpteridine, sulfadiazine, sulfamethoxazole, and L-aspartic-b-hydroxamate.

Supplementary Table 8. Complementation assay of *mdlA* in a Biolog plate

**Antibiotics**	**Concentration (mM)**	**Strains**		
		BW25113	BW25113 Δ*mdlA mdlA*	BW25113 Δ*mdlA*
Cefazolin	0.026	+++	+++	+++
Cefazolin	0.077	+++	+++	+++
Cefazolin	0.231	+++	+++	+++
Cefazolin	0.693	++	++	++
Vancomycin	0.008	+++	+++	++
Vancomycin	0.025	+++	+++	++
Vancomycin	0.076	++	++	++
Vancomycin	0.228	-	-	-

The wildtype (BW25113), the *mdlA* mutant (MG1655 **Δ***mdlA*) and the *cyuR*-complemented strain (BW25113 Δ*mdlA mdlA*) were grown in Biolog PM11C and PM12B plate. No clear differences were observed.

Supplementary Table 9. A list of oligonucleotides utilized in this study

Primer name	Sequence 5' to 3'
etp_FWD	CATGGCGTATCTCTTGAAGGACAT
etp_REV	CATTGCCCAAACAGCATTGTCT
yaiV_FWD	GAGGGAGTTATCTCTCTGCGTAGA
yaiV_REV	GGCTGGTAGATGATATCCCGTACA
rpoA_FWD	CCGAGGTTGAGATTGATGGTGTA
rpoA_REV	GTGATATCGGCTGCAGTCACA

Supplementary Figures

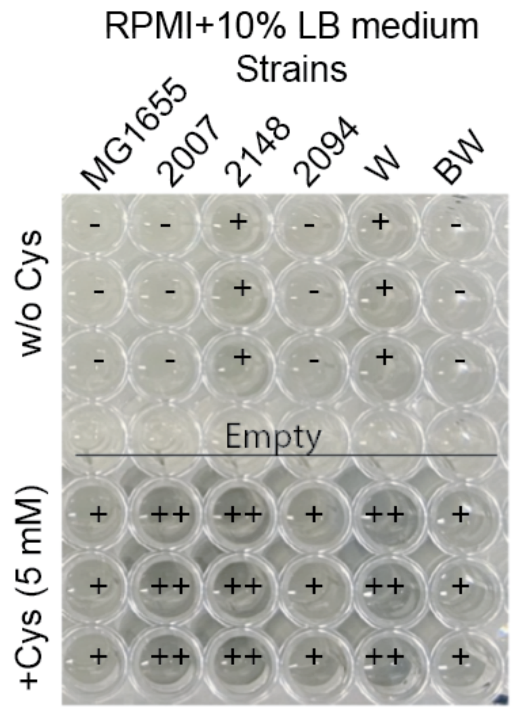

Supplementary Figure 1. Production of hydrogen sulfide by *E. coli* strains

H_2S production by grown laboratory *E. coli* strains (MG1655, W, BW25113-labeled as BW) and three clinical *E. coli* isolates (GN02007, GN02148, GN02094, labeled as 2007, 2148, 2094, respectively) in the RPMI+10% LB medium. The pictures were taken after 16 hours at 37 °C. In the photo, – (no hydrogen production), + (mild hydrogen production), ++ (high hydrogen production) indicate turbidity of each well.

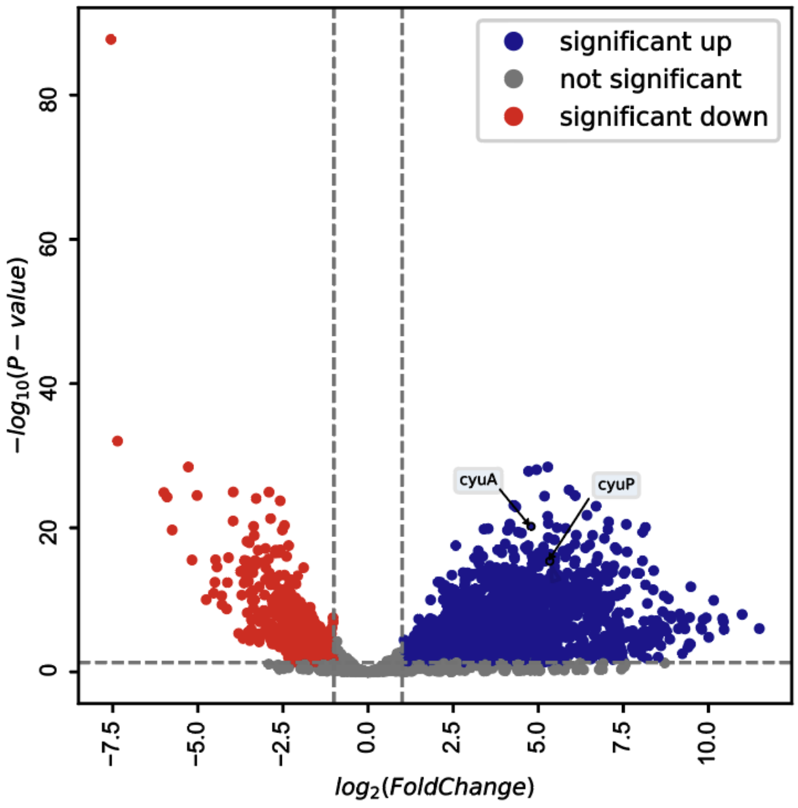

Supplementary Figure 2. Differential gene expression after the addition of 5 mM Cys

Among 4,296 annotated genes, 2,338 genes were differentially expressed where 1,644 and 694 genes were up- and down-regulated by a fold change greater than 2. Two genes, *cyuA* and *cyuP*, the two known regulation targets, were highlighted.

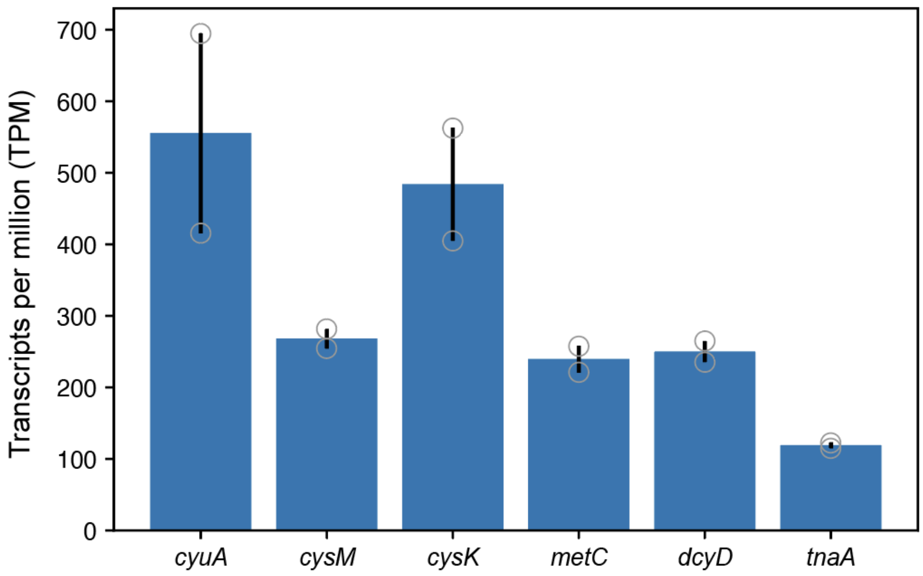

Supplementary Figure 3. Transcripts per million (TPMs) of L-cysteine desulfidase genes

x-axis and *y*-axis indicate gene names and Transcripts per million (TPM).

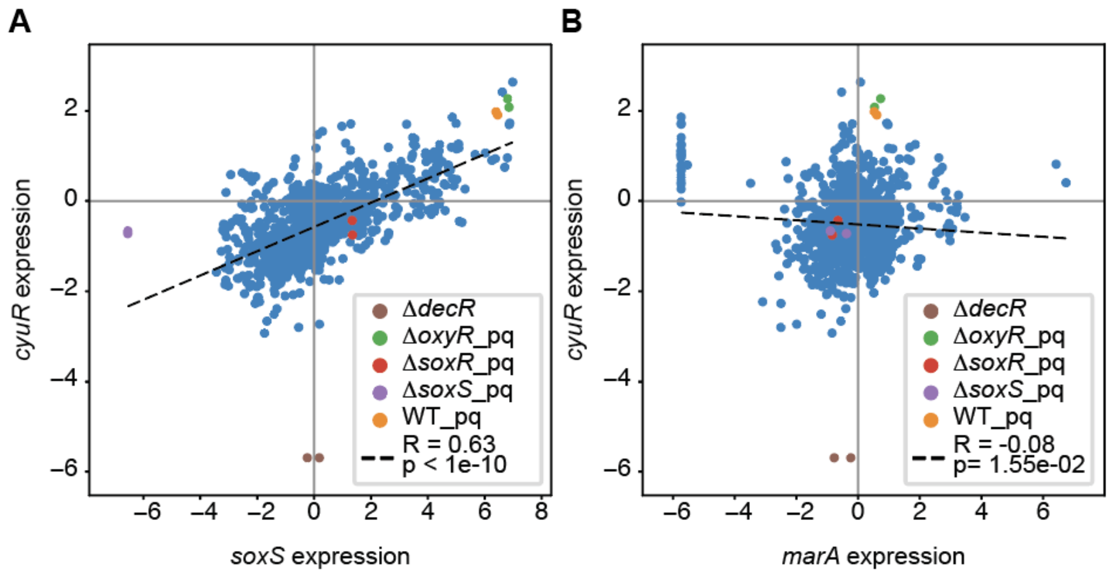

Supplementary Figure 4. Expression comparison between *cyuR* and related genes

Gene expression correlation between *cyuR* and (**A**) *soxS* and (**B**) *marA* reported in PRECISE-1K[2].

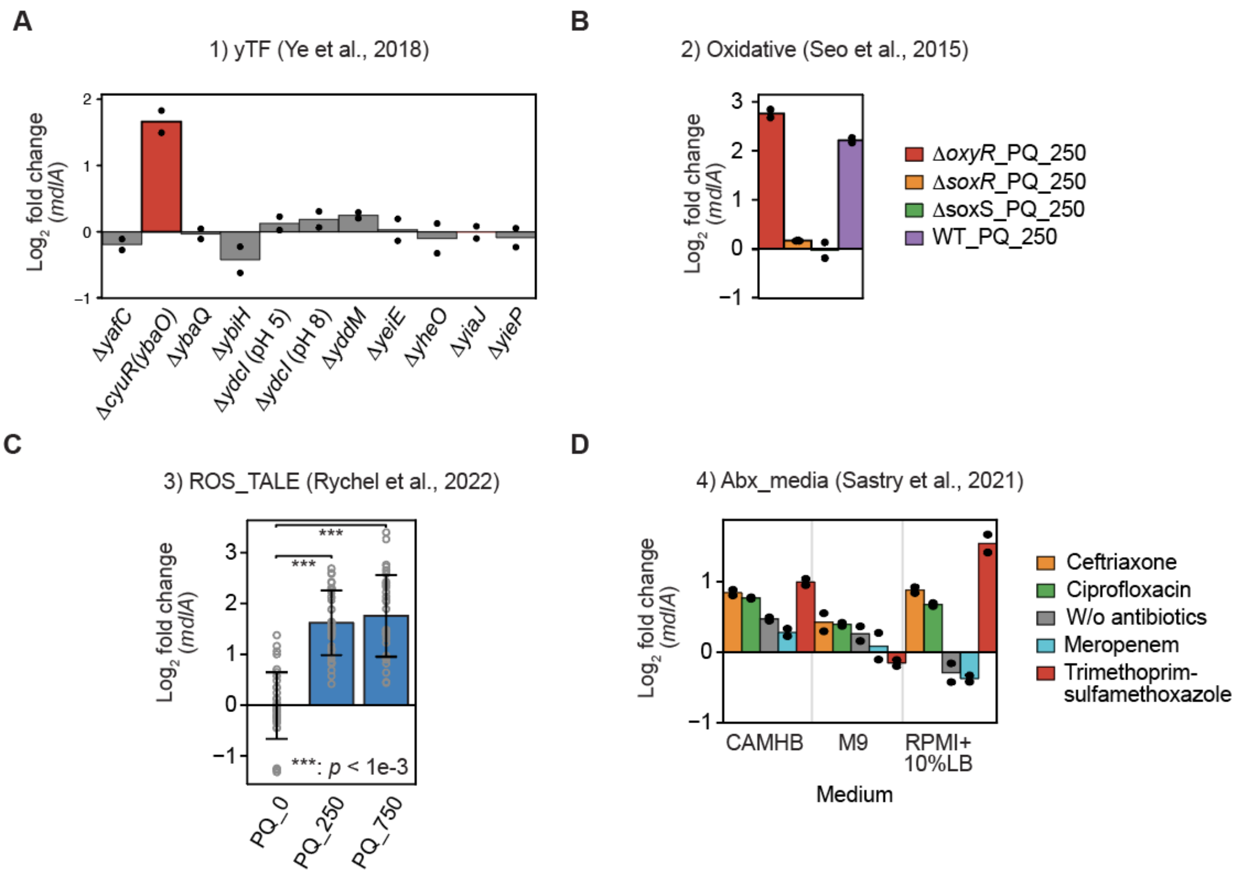

Supplementary Figure 5. *mdlA* expression levels in PRECISE-1K projects

(**A-D**) *mdlA* expression levels in samples in four projects in PRECISE-1K: yTF[3], Oxidative[4], ROS_TALE[5], Abx_media[6].

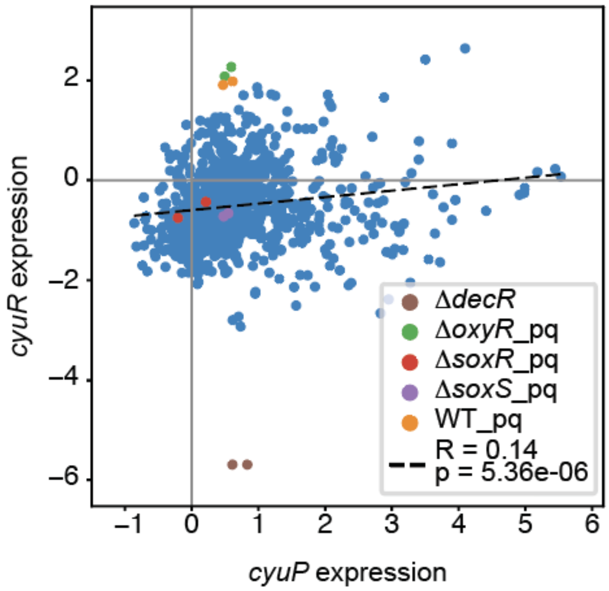

Supplementary Figure 6. Expression comparison between *cyuR* and *cyuP*

Gene expression correlation between *cyuR* and *cyuP* reported in PRECISE-1K[2].

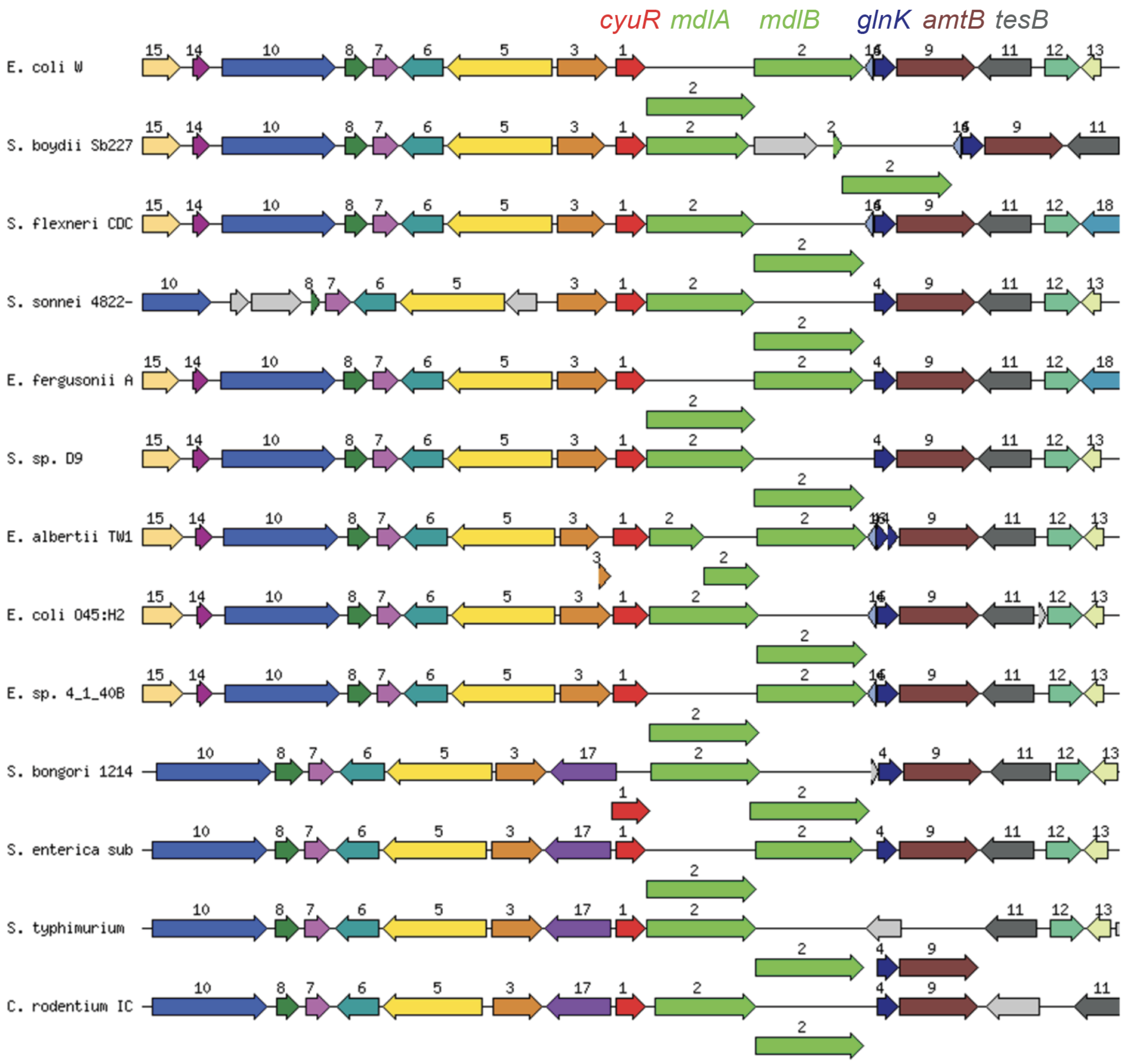

Supplementary Figure 7. Genome alignment of *cyuR-mdlAB* containing regions in multiple representative microorganisms

This figure was generated by "The SeedViewer" (https://pubseed.theseed.org). Compared microorganisms are *E. coli, E. fergusonii, Shigella flexneri, Shigella boydii, Enterobacter cloacae, Citrobacter koseri, Klebsiella oxytoca, Salmonella typhimurium, Salmonella enterica, Yersinia regensburgei, Hafnia alvei.* Examples of aligned genes: 1, *cyuR*(*ybaO*) encoding DNA-binding transcriptional activator; 2, *mdlA* encoding ABC transporter family protein MdlA or *mdlB* encoding ABC transporter family protein MdlB; 4, *glnK* encoding nitrogen regulatory protein PII-2; 9: *amtB* encoding ammonium transporter; 11, *tesB* encoding acyl-CoA thioesterase

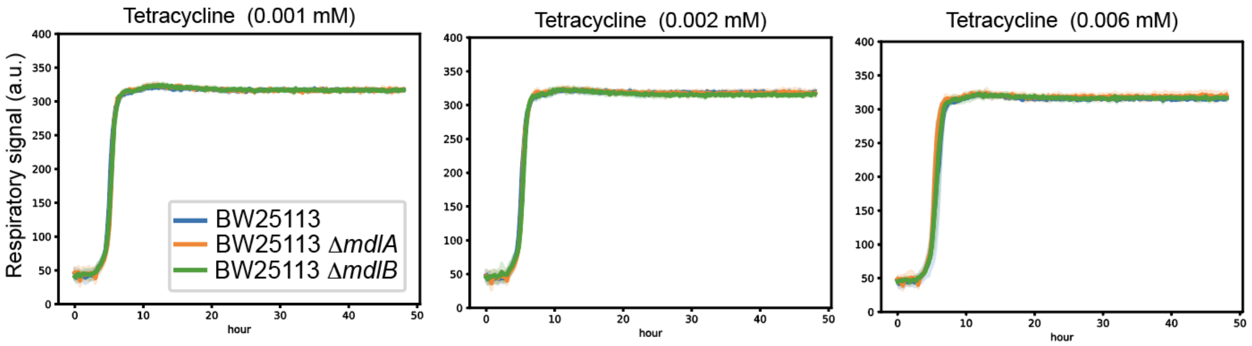

Supplementary Figure 8. Phenotype microarray data for BW25113, BW25113 Δ*mdlA*, and BW25113 Δ*mdlB* in the presence of three different concentrations of tetracycline.

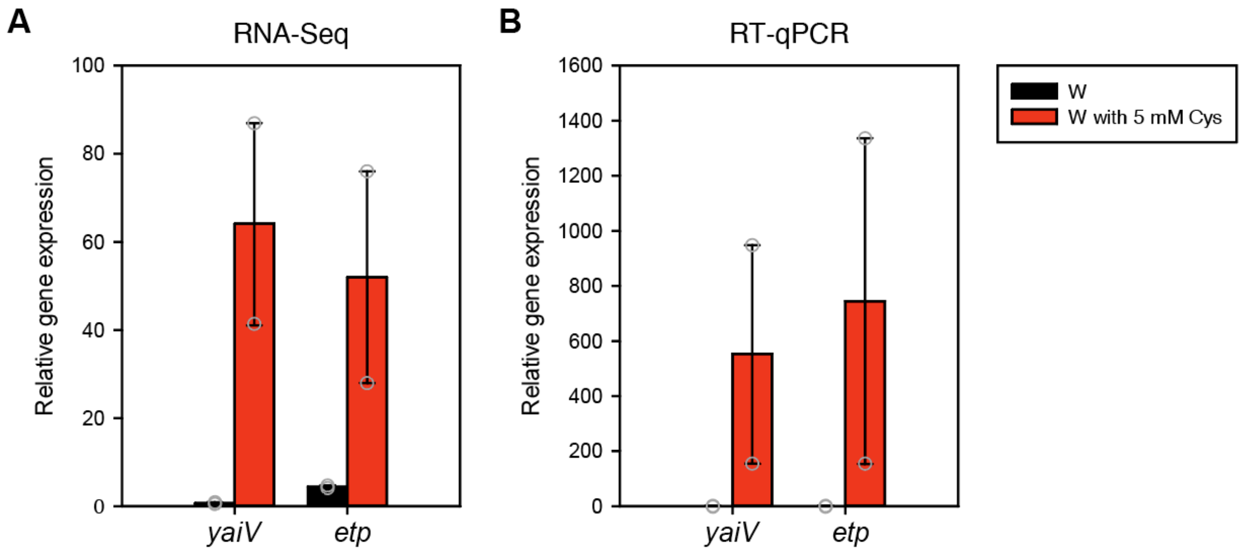

Supplementary Figure 9. Up-regulation of *yaiV* and *etp* by supplementing 5 mM Cys.

Relative expression of *yaiV* and *etp* measured by (**A**) RNA-seq and (**B**) real-time quantitative PCR (RT-qPCR).

Supplementary References

1. Sastry, A. V. *et al.* The Escherichia coli transcriptome mostly consists of independently regulated modules. *Nat. Commun.* **10**, 5536 (2019).

2. Lamoureux, C. R. *et al.* A multi-scale expression and regulation knowledge base for Escherichia coli. *Nucleic Acids Res.* (2023) doi:10.1093/nar/gkad750.

3. Gao, Y. *et al.* Systematic discovery of uncharacterized transcription factors in Escherichia coli K-12 MG1655. *Nucleic Acids Res.* **46**, 10682–10696 (2018).

4. Seo, S. W., Kim, D., Szubin, R. & Palsson, B. O. Genome-wide Reconstruction of OxyR and SoxRS Transcriptional Regulatory Networks under Oxidative Stress in Escherichia coli K-12 MG1655. *Cell Rep.* **12**, 1289–1299 (2015).

5. Rychel, K. *et al.* Laboratory evolution, transcriptomics, and modeling reveal mechanisms of paraquat tolerance. *Cell Rep.* **42**, 113105 (2023).

6. Sastry, A. V. *et al.* Machine Learning of Bacterial Transcriptomes Reveals Responses Underlying Differential Antibiotic Susceptibility. *mSphere* **6**, e0044321 (2021).

7. Baba, T. *et al.* Construction of Escherichia coli K-12 in-frame, single-gene knockout mutants: the Keio collection. *Mol. Syst. Biol.* **2**, 2006.0008 (2006).

8. Bethke, J. H. *et al.* Environmental and genetic determinants of plasmid mobility in pathogenic *Escherichia coli*. *Sci Adv* **6**, eaax3173 (2020).

9. Kitagawa, M. *et al.* Complete set of ORF clones of *Escherichia coli* ASKA library (a complete set of *E. coli* K-12 ORF archive): unique resources for biological research. *DNA Res.* **12**, 291–299 (2005).
