# Supplementary material for: CyuR is a dual regulator for L-cysteine dependent antimicrobial resistance in Escherichia coli
Source: Commun Biol. 2024 Sep 17;7:1160. doi: 10.1038/s42003-024-06831-0 (PMC11408624; doi:10.1038/s42003-024-06831-0)
Supplement: Supplementary file 2 — Description of Additional Supplementary File [file 42003_2024_6831_MOESM2_ESM.pdf]

Description Of Additional Supplementary Files

File name: Supplementary Data 1
Description: The source data for the graphs in the paper
