# Supplementary material for: CyuR is a dual regulator for L-cysteine dependent antimicrobial resistance in Escherichia coli
Source: Commun Biol. 2024 Sep 17;7:1160. doi: 10.1038/s42003-024-06831-0 (PMC11408624; doi:10.1038/s42003-024-06831-0)
Supplement: Supplementary file 4 — reporting-summary [file 42003_2024_6831_MOESM4_ESM.pdf]

nature portfolio

Corresponding author(s): Irina Rodionova & Bernhard Palsson

Last updated by author(s): Jul 17, 2024

Reporting Summary

Nature Portfolio wishes to improve the reproducibility of the work that we publish. This form provides structure for consistency and transparency in reporting. For further information on Nature Portfolio policies, see our Editorial Policies and the Editorial Policy Checklist.

Statistics

For all statistical analyses, confirm that the following items are present in the figure legend, table legend, main text, or Methods section.

n/a	Confirmed	
☐	☒	The exact sample size (*n*) for each experimental group/condition, given as a discrete number and unit of measurement
☐	☒	A statement on whether measurements were taken from distinct samples or whether the same sample was measured repeatedly
☒	☐	The statistical test(s) used AND whether they are one- or two-sided<br>*Only common tests should be described solely by name; describe more complex techniques in the Methods section.*
☒	☐	A description of all covariates tested
☒	☐	A description of any assumptions or corrections, such as tests of normality and adjustment for multiple comparisons
☒	☐	A full description of the statistical parameters including central tendency (e.g. means) or other basic estimates (e.g. regression coefficient) AND variation (e.g. standard deviation) or associated estimates of uncertainty (e.g. confidence intervals)
☒	☐	For null hypothesis testing, the test statistic (e.g. *F*, *t*, *r*) with confidence intervals, effect sizes, degrees of freedom and *P* value noted<br>*Give P values as exact values whenever suitable.*
☒	☐	For Bayesian analysis, information on the choice of priors and Markov chain Monte Carlo settings
☒	☐	For hierarchical and complex designs, identification of the appropriate level for tests and full reporting of outcomes
☐	☒	Estimates of effect sizes (e.g. Cohen's *d*, Pearson's *r*), indicating how they were calculated

Our web collection on statistics for biologists contains articles on many of the points above.

Software and code

Policy information about availability of computer code

Data collection	N/A
Data analysis	For RNAseq, raw-sequencing reads were aligned to the reference genome of the E. coli MG1655 strain (accession number: NC_000913.3) using Bowtie2. Transcripts per million calculation and differentially expressed gene analysis were performed by DESeq2 (v1.22.1). iModulon activity analysis was performed by using the Pymodulon package (https://github.com/SBRG/pymodulon).<br><br>The potential CyuR-binding site was identified by a phylogenetic footprinting approach using multiple sequence alignments. Orthologs of the E. coli cyuR gene in other Proteobacteria, as well as multiple sequence alignment of orthologous upstream region, was identified using the PubSEED comparative genomics platform. CyuR-binding site logos were obtained by using the WebLogo tool. The aligned CyuR sites from E. coli and other proteobacteria were used as a training set to build a Positional Weight Matrix (PWM) Positional nucleotide weights in the CyuR-PWM and Z-scores of candidate sites were calculated as the sum of the respective positional nucleotide weights.<br>Genome scanning for identifying additional candidate CyuR-binding sites was performed using the Genome Explorer software. The threshold for the site search was defined as the lowest score observed in the training set (Z-score = 4.5).

For manuscripts utilizing custom algorithms or software that are central to the research but not yet described in published literature, software must be made available to editors and reviewers. We strongly encourage code deposition in a community repository (e.g. GitHub). See the Nature Portfolio guidelines for submitting code & software for further information.

Data

Policy information about availability of data

All manuscripts must include a data availability statement. This statement should provide the following information, where applicable:

- Accession codes, unique identifiers, or web links for publicly available datasets
- A description of any restrictions on data availability
- For clinical datasets or third party data, please ensure that the statement adheres to our policy

The RNA-seq raw data files were uploaded to Gene Expression Omnibus (https://www.ncbi.nlm.nih.gov/geo) under an accession id, GSE215167.

Human research participants

Policy information about studies involving human research participants and Sex and Gender in Research.

Reporting on sex and gender	N/A
Population characteristics	N/A
Recruitment	N/A
Ethics oversight	N/A

Note that full information on the approval of the study protocol must also be provided in the manuscript.

Field-specific reporting

Please select the one below that is the best fit for your research. If you are not sure, read the appropriate sections before making your selection.

☒ Life sciences ☐ Behavioural & social sciences ☐ Ecological, evolutionary & environmental sciences

For a reference copy of the document with all sections, see nature.com/documents/nr-reporting-summary-flat.pdf

Life sciences study design

All studies must disclose on these points even when the disclosure is negative.

Sample size	All experiments were performed with biological duplicates for each strain shown in the figures.
Data exclusions	No data was excluded from the analysis.
Replication	All experiments were confirmed to be reproducible.
Randomization	Experimental samples were not allocated into groups. Colonies were randomly selected.
Blinding	Growth or gene expression was compared with parental strains or negative controls (i.e., untreated condition). Therefore, blinding is not necessary.

Reporting for specific materials, systems and methods

We require information from authors about some types of materials, experimental systems and methods used in many studies. Here, indicate whether each material, system or method listed is relevant to your study. If you are not sure if a list item applies to your research, read the appropriate section before selecting a response.

Materials & experimental systems

n/a	Involved in the study
☒	☐ Antibodies
☒	☐ Eukaryotic cell lines
☒	☐ Palaeontology and archaeology
☒	☐ Animals and other organisms
☒	☐ Clinical data
☒	☐ Dual use research of concern

Methods

n/a	Involved in the study
☒	☐ ChIP-seq
☒	☐ Flow cytometry
☒	☐ MRI-based neuroimaging
